# Monomeric C-reactive protein-a key molecule driving development of Alzheimer’s disease associated with brain ischaemia?

**DOI:** 10.1038/srep13281

**Published:** 2015-09-03

**Authors:** M. Slevin, S. Matou, Y. Zeinolabediny, R. Corpas, R. Weston, D. Liu, E. Boras, M. Di Napoli, E. Petcu, S. Sarroca, A. Popa-Wagner, S. Love, M. A. Font, L. A. Potempa, R. Al-baradie, C. Sanfeliu, S. Revilla, L. Badimon, J. Krupinski

**Affiliations:** 1 School of Healthcare Science, John Dalton Building, Manchester Metropolitan University, Chester Street, Manchester, M1 5GD, UK; 2 University of Medicine and Pharmacy, Targu Mures, Romania; 3 Department of Pathology/Medicine, Griffith University, Brisbane, Australia; 4 Neurological Service, San Camillo de’ Lellis General Hospital, Rieti, Italy; 5 Clinic of Neurology, Medical University Greifswald, Germany; 6 Department of Neuropathology, Institute of Clinical Neurosciences, School of Clinical Sciences, University of Bristol, Bristol, BS16 1LE, UK; 7 Acphazin, Inc., Deerfield, Illinois, USA; 8 College of Applied Medical Science, Al Majmaah University, Majmaah City, Kingdom of Saudi Arabia P.O Box 66; 9 Instituto De Investigaciones Biomedicas De Barcelona, CSIC, Barcelona, Spain; 10 CSIC-ICCC, Hospital de la Santa Creu I Sant Pau, Barcelona, Spain; 11 Hospital Universitari Mútua de Terrassa, Department of Neurology, Terrassa (Barcelona), Spain

## Abstract

Alzheimer’s disease (AD) increases dramatically in patients with ischaemic stroke. Monomeric C-reactive protein (mCRP) appears in the ECM of ischaemic tissue after stroke, associating with microvasculature, neurons and AD-plaques, Aβ, also, being able to dissociate native-CRP into inflammatory, mCRP *in vivo*. Here, mCRP injected into the hippocampal region of mice was retained within the retrosplenial tract of the dorsal 3rd ventrical and surrounding major vessels. Mice developed behavioural/cognitive deficits within 1 month, concomitant with mCRP staining within abnormal looking neurons expressing p-tau and in beta-amyloid 1-42-plaque positive regions. mCRP co-localised with CD105 in microvessels suggesting angiogenesis. Phospho-arrays/Western blotting identified signalling activation in endothelial cells and neurons through p-IRS-1, p-Tau and p-ERK1/2-which was blocked following pre-incubation with mCRP-antibody. mCRP increased vascular monolayer permeability and gap junctions, increased NCAM expression and produced haemorrhagic angiogenesis in mouse matrigel implants. mCRP induced tau244–372 aggregation and assembly *in vitro*. IHC study of human AD/stroke patients revealed co-localization of mCRP with Aβ plaques, tau-like fibrils and IRS-1/P-Tau positive neurons and high mCRP-levels spreading from infarcted core regions matched reduced expression of Aβ/Tau. mCRP may be responsible for promoting dementia after ischaemia and mCRP clearance could inform therapeutic avenues to reduce the risk of future dementia.

The incidence of dementia increases several-fold after acute ischaemic stroke^1^,^2^. There is strong evidence that brain ischaemia increases the risk of both vascular dementia and Alzheimer’s disease (AD). Most risk factors for brain ischaemia and stroke probably increase the risk of AD^3^. The reasons for this have not been clearly identified, but one possible contributor is the inflammatory response to brain ischaemia, with inflammatory mediators contributing significantly to subsequent brain damage in both AD and stroke^4^.

C-reactive protein (CRP) is pentraxin produced mainly by the liver, circulating elevated levels of which are significantly associated with AD pathophysiology, and in addition, where affected brain regions including neurons have been shown to synthesise this protein de novo^5^,^6^. There are two distinct isoforms of CRP. Native CRP is a pentameric oligoprotein and acute phase reactant, produced during active inflammatory disease at physiological circulating levels of between 40–200 μg/ml^7^. Unlike other pentraxins, native CRP can be dissociated irreversibly to form free sub-units or monomeric CRP (mCRP) which has much lower aqueous solubility, becomes tissue ECM-associated and has been shown to accumulate within tissue regions of vascular damage/inflammation, particularly, in reference to this work, accumulating in active brain neo-microvessels after ischaemic stroke^8^. This gives CRP unique properties amongst the pentraxin family. mCRP is strongly angiogenic both *in vitro* and *in vivo* and is likely to contribute to the neovascularisation of tissues in which it is deposited or synthesised^9^.

The production and characterization of antibodies capable of distinguishing between native CRP and mCRP has helped enormously in allowing us to identify biologically active mCRP in tissues^10^. To this end, recently, Strang *et al*.^11^ demonstrated that Aβ plaques generated *in vitro*, could dissociate nCRP into mCRP thus potentiating inflammatory micro-environments and promoting progression of the disease. There is strong support for the theory recently elaborated on by Marchesi *et al*.^4^ suggesting that the earliest pathological process in AD may be oxidative inflammatory damage to cerebral microvessels, provoked by peri-capillary accumulation of Aβ, leading to cerebral amyloid angiopathy (CAA) and stroke, further production of Aβ, neuronal dysfunction and damage. In support of this, Bulbarelli *et al*.^12^ demonstrated recently Aβ(42) microangiopathy and AβPP in rat brain EC exposed to glucose-oxygen deprivation, de novo synthesis of Aβ peptides by damaged brain microvessels and intracellular deposition may be a key event in developing Cerebral Amyloid Angiopathy (CAA). As yet, there is no definitive data or published work describing the effects of mCRP on neuronal function and health.

We were interested in the contribution of mCRP to the progression of AD in association with inflammation. Based on our previous findings that large quantities of mCRP are deposited in damaged brain regions from the circulation via compromised vasculature after stroke^8^, we hypothesised that this could contribute to pathological neurodegeneration. In this study, we provide strong evidence of a mechanism for dysfunctional vascular development and neuronal activation associated with mCRP. This could have implications concerning both vascular and neuronal degeneration, linked with neurodegenerative processes and in particular vascular dementia/CAA.

## Materials and Methods

### Monomeric and native CRP source

Recombinant forms of both mCRP and nCRP (0.5 mg/mL in 25 mM NaPBS, pH 7.4) was produced in the laboratory of Dr. L.A. Potempa as previously described^8^,^9^,^10^,^13^. The mCRP solution contained an endotoxin concentration lower than 0.125 EU/mL and all cell culture medium was endotoxin-free.

### Antibodies to mCRP and nCRP

Mouse monoclonal antibodies to human nCRP (N2C10), and the octapeptide of the mCRP sub-unit (M8C10) were obtained from Dr L.A. Potempa and fully characterized as described previously^8^.

### Cell culture

Bovine aortic endothelial cells (BAEC) were isolated as previously described by Sattar *et al*.^14^. BAEC were seeded in 75-cm^2^ flasks pre-coated with 0.1% gelatin (Sigma, UK) and cultured in Dulbecco’s Modified Eagle Medium (Lonza, UK) supplemented with 20% foetal bovine serum (FBS, Cambrex, UK), 2 mM glutamine and 1% antibiotics (100 μg/ml streptomycin, 100 U/ml penicillin). BAEC were placed at 37 °C in a saturated air humidity/5% CO2-incubator. Every 2–3 days, the cells were detached by enzymatic digestion with 0.05% trypsin/0.02% EDTA and split at a ratio of 1:2 or 1:3. At confluence, EC were identified by their typical cobblestone morphology and “hill and valley” configuration, respectively. The cells were used throughout the study between passages 4 and 9. Rat cortical neurons (RCN) were obtained from Life Technologies (UK) and cultured directly into T-25 flasks. These were used at the same cell concentration and under the same conditions as BAEC, and according to the manufacturer’s instructions.

### Matrigel^TM^ endothelial tube formation assay

BAEC (1.5 × 10^6^ cells/ ml) were mixed in equal volume with growth factor-reduced Matrigel^TM^ (10 mg/ml) with or without 5 μg/ml mCRP, and a spot of the mixture was poured into the centre of each well in a 48-well plates (Nunc). After polymerization of the gel for 1 h at 37 °C, each spot of cells embedded in Matrigel^TM^ was bathed in 500 μl of complete medium. After 24 h incubation, some cells migrated and aligned to form tubes (defined by the enclosure of circumscribed areas), a parameter of quantification^15^. For the counting of enclosed areas, the cells were fixed with 4% PFA for 15 min and counts made in five fields by microscopy using the ×20 objective. Using the same experimental procedure described above, BAEC were mixed with anti-mCRP antibodies (1 μg/ml) at the same time as matrigel and mCRP (5 μg/ml) to assess if the antibody was capable of blocking mCRP-induced angiogenesis. Experiments were repeated three times and results are shown as mean ± S.D. (*p < 0.05)

### Spheroid sprouting assay for junctional analysis by T.E.M

The preparation of BAEC spheroids was performed as described previously^15^. Briefly, cells were harvested from sub-confluent monolayer cultures by trypsinization and 6 × 10^5^ cells were suspended in DMEM 10% FBS and 0.25% (w/v) carboxymethylcellulose (Sigma, UK). Cells were then seeded into non-adherent round-bottom 96-well plates to assemble into a single spheroid within 24 h at 37 °C, 5% CO_2_. Basic fibroblast growth factor (human recombinant FGF-2, (BD Bioscience, UK)) was used as positive control and added at a final concentration of 25 ng/ml. After 24 h, gels were photographed and sprouting architecture was assessed microscopically. Experiments were performed in triplicate.

### Endothelial cell permeability assay

BAEC were cultured to confluence on Transwell collagen coated permeable (pored) supports (Millipore) and medium replaced with SPM (24 h). mCRP (10 μg/ml) or DMSO (1 μg/ml; positive control) was added and cells incubated at 37 °C for a further 8 h. Next, the medium was refreshed and replaced with 500 μl of basal medium and a solution of FITC-dextran (150 μl; 1:20 added to each well and incubation continued at room temperature for a further 4 h. The quantity of FITC-dextran passing through the pores of the insert into the collecting plate was proportional to the permeability of the monolayer. 100 μl of the collecting medium was read on a fluorescent plate reader with filters at 485 and 535 nm excitation and emission according to the manufacturer’s instructions. Photomicrographs of the monolayers stained with Millipore cell stain (10 minutes) were also obtained. Each test was performed in triplicate and the experiment performed twice, a representative example is shown and p < 0.01 is indicated by *).

### Mouse matrigel implant

In this pilot study (n = 3 per group), aliquots of growth factor reduced matrigel (400 μl) were injected into C57BL/6 mice subcutaneously at the dorsal surface,+/− addition of VEGF (positive control 10 μg/ml) or nCRP/mCRP (10 μg/ml). After 5 days, the animals were euthanized and plugs dissected and photographed in order to identify evidence of vascularization and neo-circulation. Simple statistical analysis (mean ± S.D. was used to count the numbers of vessels associated with each implant. Detailed histological analysis of the plugs was not carried out.

### Short-Interfering RNA (siRNA) targeting IRS-1

Short-interfering RNA duplexes targeting bovine IRS1 (IRS1 siRNA) and negative control (NC siRNA) were selected and chemically synthesised by GenePharma (Shanghai, China). Their sequences were:

5′ GCGGUAGUGGCAAACUCUUTT 3′(sense) 5′ AAGAGUUUGCCACUACCGCTT 3′ (anti-sense)

To examine the transfection efficiency of Lipofectamine2000 (Invitrogen), NC-FAM (negative control-fluorescein amino-modified oligonucleotides) siRNA was transfected into BAEC previously seeded on glass coverslips, and the following day the cells were washed with PBS, fixed with 4% PFA and visualised with a Zeiss fluorescent microscope. For mRNA ‘knock-down’, the reverse transfection method was applied as described by Elbashir *et al*.^16^. Briefly, the siRNA duplexes were transfected into 70–80% confluent BAEC cultured in a 24-well plate at a final concentration of 50 nM. The cells were incubated at 37 °C for 2 hours before the addition of 10% FBS with 100 U/ml penicillin and 100 μg/ml streptomycin then cultured for a further 24 h for RT-PCR, tube-formation analysis. Experiments were repeated three times and results shown as the mean ± S.D (*p < 0.05).

### RNA extraction and RT-PCR

Extraction of total RNA from BAEC was performed and complementary DNA (cDNA) was produced from total RNA extracts in two stages as described by the standard protocols and subsequently used to monitor bovine IRS1 mRNA expression. The gene-specific primer pairs used for PCR were optimized for cycle number (IRS1 = 35 cycles) and t_m_ (58 °C). Primer pairs (Invitrogen) were selected by software Primer3 and the sequences are shown above. PCR products were analysed by 1.5% agarose gel electrophoresis. Ribosomal protein S14 expression was used as an internal standard.

### Kinexus phospho-protein array analysis

BAEC or RCN were seeded in 6-well plates at a concentration of 3 × 10^5^ cells/2 ml in complete medium. After 48 h incubation, the medium was replaced with serum-free medium for a further 24 h incubation then 5 μg/ml mCRP was added to the cells and allowed to incubate for 8 min at 37 °C. To screen the modulation of the signalling protein expression downstream of the mCRP receptor, a phospho-protein micro-array analysis of 500 phospho-site proteins, Kinex^TM^ Antibody Microarray (KAM)-1.2 was performed by Kinexus Bioinformatics (Vancouver, Canada). Proteins from mCRP-stimulated and un-stimulated cells were extracted according to Kinetworks instructions. Briefly, cells were washed twice with cold PBS. Protein was extracted using a buffer (pH 7.2) containing 20 mM MOPS, 2 mM EGTA, 5 mM EDTA, 30 mM sodium fluoride, 60 mM β-glycerophosphate, 20 mM sodium pyrophosphate, 1 mM sodium orthovanadate, 1 mM phenylmethylsulfonylfluoride, 3 mM benzamidine, 5 μM pepstatin A, 10 μM leupeptin, 1% Triton X-100 and 1 mM dithiothreitol on ice for cell lysis. Cell extracts were then collected and protein concentrations were measured by the Bradford protein assay using a plate reader and according to the manufacturer’s instructions (Bio-rad, Munchen, Germany). For each sample, protein (100 μg in 50 μl) was transferred to a fresh 1.5 ml Eppendorf screw cap and this was sent to Kinexus for analysis.

### Western blot Analysis

BAEC/RCN were seeded in complete medium in a 24-well plate at a cell concentration of 10^5^/ml/well. After 48 h incubation, the medium was renewed with SPM and cells incubated for a further 24 h Next, 5 μg/ml mCRP was added and the cells incubated for 8 min at 37 °C. In addition, BAEC cultured under the same conditions were exposed to mCRP antibody (1 μg/ml) immediately prior to addition of mCRP in some experiments in order to identify any potential blocking effects on cell signalling. After rapid washing in cold PBS, cells were lysed with 120 μl/well of ice-cold radioimmunoprecipitation (RIPA) buffer (pH 7.5) containing 25 mM Tris-HCl, 150 mM NaCl, 0.5% sodium deoxycholate, 0.5% SDS, 1 mM EDTA, 1 mM sodium orthovanadate (EGTA), 1 mM phenylmethylsulfonyl fluoride (PMSF), 1% Triton X100 and 1 μM leupeptin. The protein concentration of cell lysates was determined using the Bradford protein assay (Bio-rad, Munchen, Germany) and equal quantities of proteins (15 μg) were mixed with 2 X Laemmli sample buffer, boiled in a water bath for 15 min then centrifuged. Samples were separated along with pre-stained molecular weight markers (32,000–200,000 Da) by 12% SDS-PAGE. Proteins were electroblotted (Hoefer, Bucks, UK) onto nitrocellulose filters (1 h) and the filters were blocked for 1 h at room temperature in TBS-Tween (pH 7.4) containing 1% bovine serum albumin (BSA). Filters were stained with the following primary antibodies diluted in the blocking buffer, overnight at 4 °C on a rotating shaker: rabbit monoclonal antibodies to phospho-IRS1 (Y1179), rabbit polyclonal antibodies to γ-secretase subunit presenilin enhancer protein 2 (1:1000) from Acris Antibodies (San Diego, CA, USA), p-Tau (S404), NCAM, (1:1000), p-APP (Y757), PEN-2, p-ERK1/2 and β-amyloid (1–42; Abcam, UK; in this case, cells were cultured with mCRP for 24 h) and mouse monoclonal antibodies to β-tubulin (1:1000) from Santa Cruz Biotechnology. After washing (5 × 10 min in TBS-Tween at room temperature), filters were stained with either goat anti-rabbit or rabbit anti-mouse horse-radish peroxidase-conjugated secondary antibodies diluted in TBS-Tween containing 5% de-fatted milk (1:1000, 1 h, room temperature) with continuous mixing. After a further five washes in TBS-Tween, proteins were visualized using ECL chemi-luminescent detection (Geneflow) and semi-quantitatively identified fold differences compared with house-keeping controls determined using Image-J software. All experiments were repeated at least twice and a representative example is shown.

### NFT- Aggregation of Tau (human protein, Sigma)

The aggregation of Tau_244–372_ (8 μM) was induced by 150 μM of arachidonic acid (Sigma) in buffer containing 10 mM Hepes (pH 7.6), 100 mM NaCl, 5 mM dithiothreitol for 24 h incubation at room temperature without stirring^16^. To detect a direct effect of mCRP on Tau aggregation, Tau was incubated with mCRP (10 μg/ml) in the same buffer (minus arachidonic acid) for 24 h (4 h-5 days pilot study estimates were carried out to optimise this-data not included) at room temperature. The protein samples were fixed with 2% glutaraldehyde (Sigma), ultracentrifuged at 100,000 g for 30 minutes then spread on glass coverslips and allowed to dry in air. The samples were then sputter coated with gold and examined in a Jeol JSM-5600LV scanning electron microscope at an accelerating voltage of 12 kV and 10,000× magnification. Experiments were carried out at least twice and a representative example is shown.

### Animals

Twenty four male 3xTg-AD mice and twenty four non-transgenic (NTg) mice were used in this study. The 3xTg-AD mouse model (primarily used here as a positive AD-like control) was genetically engineered at the University of California, Irvine to express the familial AD mutations PS1/M146V, APPswe and tauP301L^17^. The NTg mice had the same genetic background hybrid 129× C57BL6 as 3xTg-AD. Mice were bred from the Spanish colony established in the Medical Psychology Unit, Autonomous University of Barcelona^18^. Genotypes were confirmed by polymerase chain reaction (PCR) analysis of DNA obtained from ear punches. Animals were individually housed in Macrolon cages (Techniplast, Buguggiatta, Italy) with free access to food and water and maintained in a temperature controlled room (22 ± 2 °C) with 12 hours light/12 hours dark cycle. Animal handling, including surgical procedures, behavioral testing and necropsies, was performed at the facilities of the Animal Unit of the University of Barcelona, Spain.

The study was approved by the local animal experimentation ethics committee (Ref: DAAM-6991, CEEA, UB). All procedures were carried out in accordance with approved Spanish guidelines/legislation concerning the protection of animals used for experimental and other scientific purposes and the European Commission Council Directive 86/609/EEC on this subject. All experimental protocols were approved by the above authority.

### Hippocampal injection of mCRP

mCRP, was delivered into the CA1 region of the mouse hippocampus by stereotactic surgery procedures. Four-month old mice (n = 8 per group) were anesthetized with 10 mg/kg xylacine (Rompun 2%, Bayer, Leverkusen, Germany) i.p. and 80 mg/kg ketamine (Ketolar 50 mg/ml, Pfizer, Alcobendas, Madrid, Spain) i.p. and placed in a stereotactic apparatus (David Kopf Instruments, Tujunga, CA). Bilateral infusions of either an experimental agent solution or artificial CSF (NaCl 148 mmol/l, KCl 3 mmol/l, CaCl2 1 mmol/l, MgCl2 0.8 mmol/l, Na2HPO4 0.8 mmol/l, NaH2PO4 0.2 mmol/l) were performed into the CA1 area of the hippocampus. Solutions of mCRP contained a final content of 50 μg. Injections were performed at a rate of 1 μl/min at coordinates relative to Bregma of −2.0 mm A/P, ± 1.2 mm M/L, −1.5 mm V/D. One microliter of the testing solutions was delivered to the application point with a 25-gauge stainless steel cannula (Small Parts Inc., Miami, FL) connected to a Hamilton syringe through a Teflon tube. The syringe was attached to a micro-infusion pump (Bioanalytical systems Inc., West Lafayette, IN). The cannula was left in position for 5 min after delivery to prevent the solution from surging back. One animal died immediately after surgery; otherwise, eight mice formed the experimental groups.

### Behavioral testing

Animals were tested for changes of non-cognitive and cognitive behavior at two-three weeks after hippocampal injections. A battery of tests was applied on fourteen daily consecutive sessions.

Sensorimotor responses: Visual reflex and posterior legs extension reflex were measured by holding the animal by its tail and slowly lowering it towards a black surface. Motor coordination and equilibrium were assessed by the distance covered and the latency to fall off a horizontal wooden rod and a metal wire rod, as described previously^19^. Prehensility and motor coordination were measured as the distance covered on the wire hang test, which consisted of allowing the animal to cling from the middle of a horizontal wire (2 mm diameter × 40 cm length) with its forepaws for two trials of 5 s and a third 60 s trial.

Corner test: Neophobia to a new home-cage was assessed by introducing the animal into the center of a standard square cage (Macrolon, 35 × 35 × 25 cm) with fresh bedding and counting the number of corners visited and rearings during a period of 30 s. The latency of the first rearing was also recorded.

Open field test: Mice were placed in the center of the apparatus (home-made, wooden, white, 55 × 55 × 25 cm high) and observed for 5 min. Patterns of horizontal locomotor activity (distance covered and thigmotaxis) and vertical movement (rearings) were analyzed throughout the test. Initial freezing, self-grooming behavior and the number of urine spots and defecation boli were also recorded.

Dark and light box test: Anxiety-like behavior was measured in a dark-light box. The apparatus consisted of two compartments (black: 27 × 18 × 27 cm with a red light; white: 27 × 27 × 27 cm with white lighting intensity of 600 lux) connected by an opening (7 × 7 cm). The mice were introduced into the black compartment and observed for 5 min. The latency to enter the lit compartment, the time spent in the lit compartment and the number of rearings were recorded.

Boissier’s four hole-board test: Exploratory behavior was measured as the number of head-dips and time spent head-dipping on each of the four holes (3 cm diameter) equally spaced in the floor of the hole-board (woodwork white box of 32 × 32 × 32 cm). The latencies of movement, first dipping and four hole dipping were recorded.

Tail suspension test: Mice were suspended by the tail to assess depression-like behavior. The mouse was hanged 30 cm above the surface. The tail was fixed with adhesive tape at 1 cm from its tip. The duration of immobility (defined as the absence of all movement except for those required for respiration) was scored during 6 min.

Object recognition test: Animals were placed in the middle of a black maze with two arms angled 90°, each measuring 25 cm × 5 cm. The 20 cm high walls could be lifted off for easy cleaning. The lighting intensity was 30 lux. The objects to be discriminated were made of wood (5–6 cm high, brightly colored). After two previous days of habituation, the animals were submitted to a 10 min acquisition trial (first trial) during which the mouse was placed in the maze in the presence of two identical novel objects (A+A’) placed at the end of each arm. A 10 min retention trial (second trial) occurred 2 h later, replacing object A’ in the maze by object B. Another 10 min retention trial (third trial) occurred 24 h later, replacing object A in the maze by object C. The time that the animal explored the new object and the old object were recorded. In order to avoid object preference biases, the sequence of presentation of the different objects was counter-balanced in each experimental group. The maze and the objects were cleaned with 96° ethanol between different animals, to eliminate olfactory cues.

Morris water maze test: Animals were tested for spatial learning and memory in the Morris water maze (MWM), consisting of one day of cue learning and six days of place task learning for spatial reference memory, followed by one probe trial. To test the spatial learning acquisition, mice were trained to locate a hidden platform, 10 cm in diameter, located 20 cm from the wall and 0.5 cm below the water surface. This was placed in a circular pool 100 cm in diameter, 40 cm height, with 25 °C opaque water, surrounded by black curtains. The animals learned to find the platform using distinctive landmarks as visual cues (four trial sessions of 60 s per day). For details of the procedure see: García-Mesa *et al*.^19^. On day seventh, after one trial of place learning, the platform was removed and the mice performed a probe trial of 60 s to test the retention of learning. A computerized tracking system (SMART, Panlab S.A., Barcelona, Spain) allowed to measure the distance covered during the learning tasks, along with the time spent in each quadrant of the pool after the removal of the platform in the probe test. For statistical analysis, 2-way ANOVA was used and significance was defined as *p < 0.05.

### Histological analysis

After completion of the behavioral tests, at 1 month after injection, mice were anesthetized as described above and transcardially perfused with 100 mM phosphate buffer (PB, pH 7.4) containing 0.1 mg/ml heparin (Mayne Pharma, Spain) followed by 4% paraformaldehyde in PB. Brains were removed and post-fixed overnight in cold paraformaldehyde, rinsed with cold PB and then dehydrated in a graded ethanol series, cleared in xylene and embedded in paraffin. Serial sections were cut throughout the brain at 5 μ, and IHC was carried out at 1 mm intervals throughout the brain-details described below in the section on immunohistochemistry (animals n = 5 per group) in order to determine expression and localization of mCRP, p-Tau, p-IRS-1 and Aβ.

### Anatomo-pathological study

Brain samples: 1) Ischaemic stroke with AD: Samples were obtained from the Institute of Neuropathology Brain Bank, University Hospital of Bellvitge, Catalonia and ethical approval for the work was granted. The tissue samples had been collected within 4 hours of death from the refrigerated bodies of 10 patients who died 2–53 days after stroke following middle cerebral artery occlusion (details are provided in Table 1). Patients presented with dementia, confirmed in memory clinics and the diagnosis of probable AD was confirmed by anatomopathology. All the AD cases had a history of progressive dementia and were selected on the basis of a diagnosis according to CERAD of ‘definite AD’^20^ and a Braak tangle stage of V-VI^21^; according to NIA-Alzheimer’s Association guidelines^22^, AD neuropathological change was considered a sufficient explanation for the dementia in all cases. Samples were dissected into infarcted (identified with 2, 3, 5-triphenyltetrazolium chloride), peri-infarcted and normal looking unaffected tissue as previously described^23^. Peri-infarcted tissue showed structural integrity but was characterised by oedema, altered morphology of the neurons (some showing changes of apoptosis), and angiogenesis. Tissue from the contralateral hemisphere served as a control. Samples were dissected into 2 mm diameter pieces and either frozen in liquid nitrogen at -70°or fixed in 10% buffered saline prior to paraffin embedding.

2) Alzheimer’s tissue (post mortem) from 20 patients and 10 negative controls was obtained from the Brain Bank in Bristol (UK). Tissue sections were obtained from blocks of the cerebral cortex, specifically, from the frontal lobe, parietal lobe and occipital lobe. Details are shown in Table 2. The AD cases all had a history of progressive dementia and were selected on the basis of a diagnosis according to CERAD of ‘definite AD’^20^ and a Braak tangle stage of V-VI^21^; according to NIA-Alzheimer’s Association guidelines^22^, AD neuropathological change was considered a sufficient explanation for the dementia in all cases. The normal controls had no history of dementia, few or no neuritic plaques, and no other neuropathological abnormalities

### Immunohistochemistry

Double immunofluorescence and/or immunohistochemistry were used to assess the distribution of mCRP (mouse anti-human mCRP-specific antibodies 8C10 and p-IRS-1-Y1179) and activated microvessels (CD105/endoglin rabbit polyclonal antibody) as well as the presence of β-amyloid and p-tau (rabbit polyclonal antibodies). After incubation with primary antibodies for 1 h at room temperature (1:100), sections were washed and then incubated with the appropriate secondary antibodies (1:50) – peroxidase (HRP), fluorescein isothiocyanate-conjugated sheep anti-mouse IgG (Jackson) or tetramethylrhodamineisothiocyanate-conjugated rabbit anti-goat (Jackson). Images were captured with Nikon 80i Digital Microscope using Nis Elements 3.21 software with multichannel capture option. Negative control slides were included where the primary antibody was replaced with PBS. Vecor ABC kits were used for all IHC and the Vector mouse on mouse (M.O.M) was used when applying mouse primary CRP antibodies to the murine brain sections with mouse secondary.

### Statistical analysis

All *in vitro* experiments were performed at least twice and where appropriate, SPSS package was used with a student t test and ANOVA to determine statistical differences from minimum of n = 3 groupings of test versus control. For the animal experiments, based on the advice of a medical statistician and the experience of behavioural studies of our team, n = 8 was used for each group of mice providing the minimum number to allow significant data to be recognized.

### Ethics statement

The mice were bred from the Spanish colony established in the Medical Psychology Unit, Autonomous University of Barcelona^18^. Genotypes were confirmed by polymerase chain reaction (PCR) analysis of DNA obtained from ear punches. Animals were individually housed in Macrolon cages (Techniplast, Buguggiatta, Italy) with free access to food and water and maintained in a temperature controlled room (22 ± 2 °C) with 12 hours light/12 hours dark cycle. Animal handling, including surgical procedures, behavioral testing and necropsies, was performed at the facilities of the Animal Unit of the University of Barcelona, Spain. The study was approved by the local animal experimentation ethics committee (Ref: DAAM-6991, CEEA, UB). All procedures were carried out in accordance with approved Spanish guidelines/legislation concerning the protection of animals used for experimental and other scientific purposes and the European Commission Council Directive 86/609/EEC on this subject. All experimental protocols were approved by the above authority. Regarding the human study, the institutional review board and local ethical committee (CEIC) of the Hospital Universitari Mútua Terrassa provided clearance for the study. All patients signed informed consent.

## Results

### Kinexus quantitative phospho-protein screens demonstrated that mCRP increased phosphorylation of Tau and IRS-1 in BAEC

**Figure 1:**
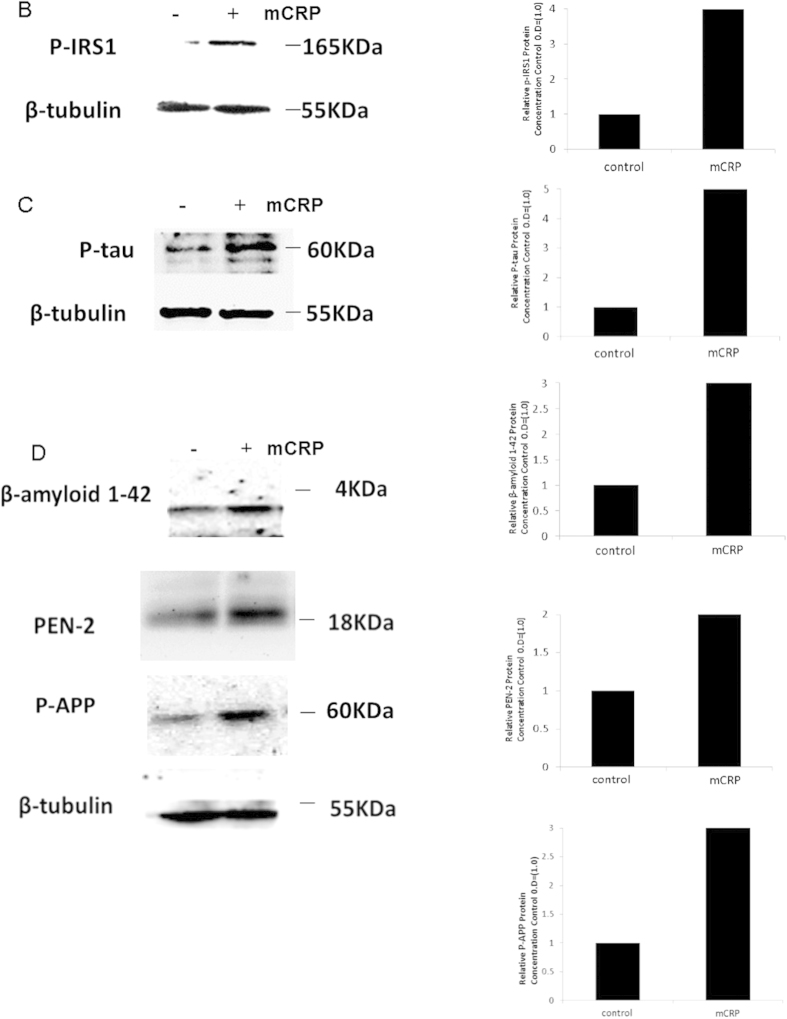
Kinexus Western phospho-microarray analysis and Western blotting of mCRP-induced signalling in BAEC. A shows quantitative Kinexus phospho-protein screening array carried out on BAEC after exposure to mCRP (8 minutes) demonstrated up-regulation of several potentially important proteins that may be implicated in AD pathology including Tau (2.3 fold) Focal adhesion kinase and IRS-1 (3.4 fold). IRS-1 was investigated in more detail in our *in vitro* studies Fig. 1B shows by Western blotting in the same samples, that mCRP induced approximately a fourfold increase in p-IRS expression compared with control untreated cells (bar chart). P-Tau was also increased by approximately 5-fold (**C**) and in addition, we showed that the cellular content of Aβ1–42 increased 3-fold over 24 h whilst PEN-2 also increased (2-fold; 8 minutes). These experiments were carried out at least twice and a representative example is shown.

We performed a Western phospho-protein screen on BAEC exposed to mCRP (10 μg/ml, 8 minutes; based on our previous published findings of maximal acute phosphorylation induced by mCRP). Results demonstrated that Tau was phosphorylated (S516) by mCRP (>2 fold) and also IRS-1 (Y1179) (>3 fold) amongst other proteins including focal adhesion kinase and Bcl2 (Fig. 1A). Western blotting confirmed the results of the kinexus screen showing that IRS-1 and tau were phosphorylated in the presence of mCRP in BAEC after 8 minutes. Approximately a 4.5 fold increase in p-IRS-1 was found in BAEC exposed to mCRP for 8 minutes (Fig. 1B), and p-tau increased by approximately 4.2 fold (Fig. 1C). The bar chart demonstrates the increase compared with control, untreated cells using β-tubulin as a house-keeping control. Since increased Tau phosphorylation, tangle formation and abnormal amyloid processing may be linked to vascular dysfunction in endothelium^24^,^25^, we went on to examine if mCRP could affect/induce NFT formation, β-amyloid 1–42 cleavage or γ-secretase-presenilin expression in BAEC. The cleaved amyloid fragment (1–42) was increased in samples (intracellular) treated with mCRP (5 μg/ml/24 h) as shown by Western blotting (2.8 fold) (Fig. 1D). Extracellular levels of amyloid-β (1–42) were not significantly altered as measured in the medium (data not shown). γ-secretase active sub-unit (presenilin enhancer protein 2; PEN-2) and phosphorylated amyloid precursor protein (p-APP) expression was also increased around 2.5 fold after 8 minutes treatment (Fig. 1D) indicating a potential mechanism for amyloid cleavage. mCRP also phosphorylated ERK and AKT as shown previously (data not included;^13^).

### Down-regulation of IRS-1 with siRNA significantly inhibited the ability of mCRP to induce angiogenesis in BAEC

**Figure 2:**
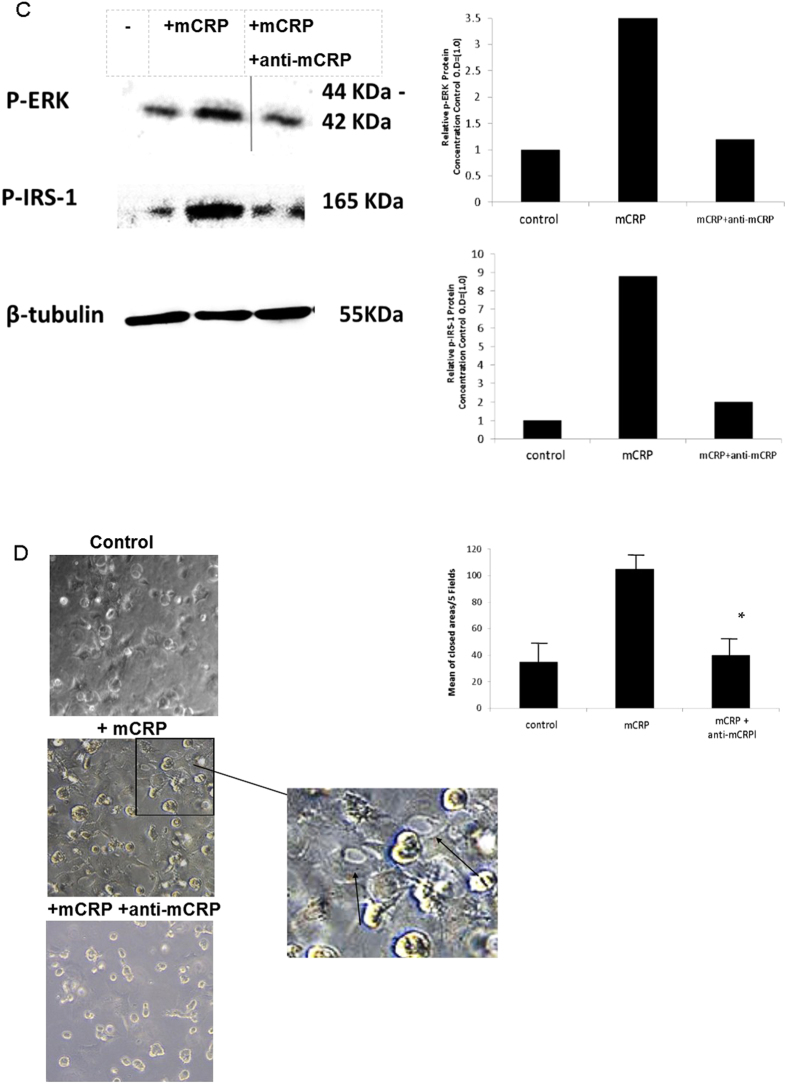
Effects of siRNA knock-down of IRS-1 on BAEC angiogenesis and cell signalling: BAEC were subjected to siRNA knock-down of IRS-1as described in the Materials and Methods section of this article. After 48 h treatment, approximately an 85% reduction in IRS-1 gene expression was noted (**A**). In contrast, NC siRNA had no effect on IRS-1 gene expression. Knock-down was tested for each experiment and found to be similar and the figure shows a representative example. (**B**) a reduction (50%) in mCRP-induced tube-like-structure formation was seen in siRNA-treated cells. The bar chart shows significant reduction in tube formation in the presence of IRS-1 siRNA (**p < 0.01; *p < 0.05). Pre-incubation with our characterised antibody specific for mCRP (4 h; 1 μg/ml) was able to inhibit both p-ERK1/2 and p-IRS-1 expression in BAEC (**C**) and also, significantly, tube-like-structure formation (**D**; *p < 0.05). Note nCRP was not tested in these assays as we have previously shown that it has no pro-angiogenic activity^8^ These experiments were carried out three times and where statistical analysis was performed, results represent the mean ± S.D of these experiments.

To confirm that IRS-1 was important in mediating the angiogenic properties of mCRP, we down-regulated IRS-1 (>85%; Fig. 2A) using siRNA and this reduced the ability of mCRP to induce tube formation in a matrigel substrate by approximately 50% (Fig. 2B; *p < 0.05 and **p < 0.01). Thus mCRP appears to operate through a pathway involving IRS-1 in order to mediate its pro-angiogenic activities (see proposed signalling pathway; Supp. Fig. 3). Similarly, addition of our mCRP antibodies (1 μg/ml) prior to addition of mCRP inhibited signalling through MAP kinase (ERK1/2 and IRS-1) and also perturbed angiogenesis following analysis of mCRP-induced tube-like structure formation, whilst the control IgG antibody had no effect (data not included) (Fig. 2C,D respectively). Note nCRP was not tested in these assays as we have previously shown that it has no pro-angiogenic activity^8^.

### mCRP induced increased vascular permeability with instability of cell-cell junctions and haemorrhagic angiogenesis *in vivo*

**Figure 3:**
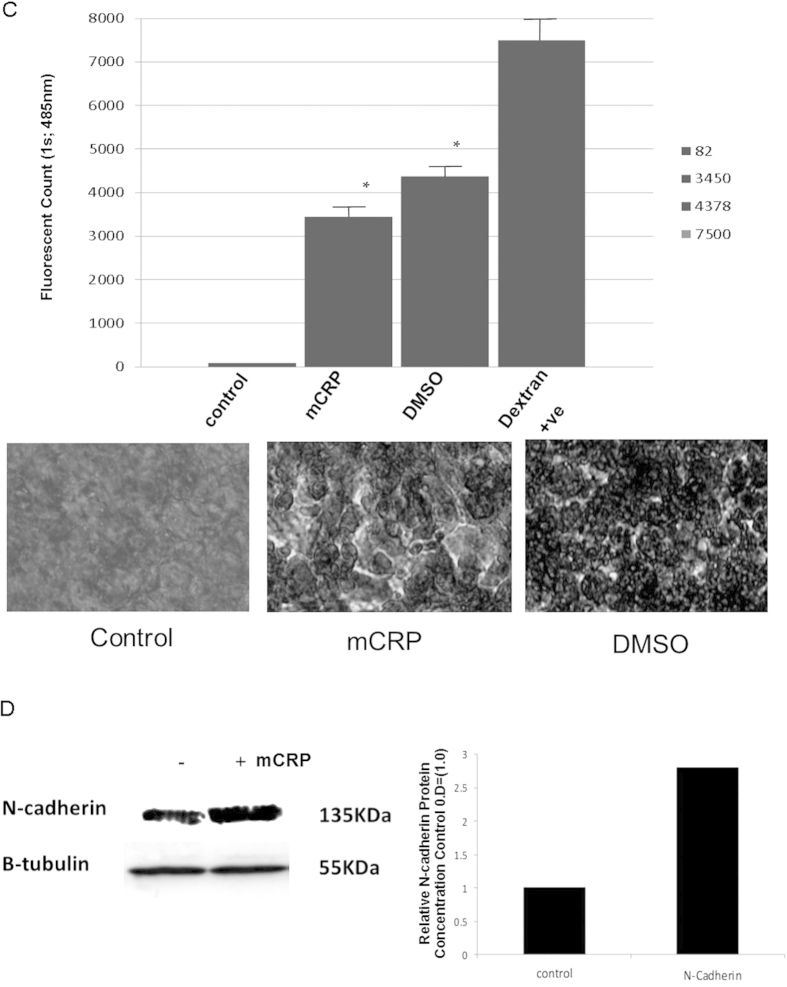
Characterization of mCRP-induced vascular activation: (**A**) BAEC spheroids were generated to examine the effect of mCRP on sprout structure and formation in a 3-dimensional system. In normal culture conditions, sprouting was slower, sprouts had a thicker appearance and cell-cell junctions were maintained (left panel). In the presence of mCRP, sprouts formed more quickly, were notably thinner in appearance and the inter-cellular gaps between cells was notably larger (right panel). (**B**) Dorsal matrigel implants containing mCRP (10 μg/ml; 72 h) produced strong and visible haemorrhagic angiogenesis (iv; arrows) compared with a typical, normal looking vascular response seen in the presence of VEGF (ii; 25 ng/ml), whilst nCRP (10 μg/ml) produced very little angiogenic response (p < 0.05 increase in the presence of mCRP and VEGF compared with control implants) (iii). In (**C**) the graph shows a significant increase in monolayer permeability in the presence of mCRP (10 μg/ml; 8 h) using a Millipore-based filter assay, similar to that produced by 10% DMSO (p < 0.01 increase in FITC dextran penetrating the monolayer in the presence of either mCRP or the positive control DMSO), and lighter regions in the images shown indicate areas of increased permeability. (**D**) Expression of adhesion molecules was examined in BAEC treated with mCRP (10 μg/ml; 24 h). NCAM expression was increased by approximately 2.8 fold whilst VCAM, ICAM and integrins were not affected (data not shown). β-tubulin was used as the house keeping control (gel and bar chart shown). These experiments were repeated at least twice and a representative example is shown.

Electron microscope images of tube-like-structures produced in matrigel from a spheroid assay, in the presence of mCRP (10 μg/ml; 24 h) showed increased gaps between adjacent cells suggesting a possible increase in vascular permeability (Fig. 3A). In addition, sprouts formed in the presence of mCRP were longer and thinner than those cultured in complete medium (detailed data published elsewhere). Changes to the structure and/or organisation of the blood vessels was confirmed *in vivo*, with mCRP (10 μg/ml applied to dorsal matrigel implants, inducing haemorrhage after 72 h, unlike the normal pro-vascularization seen in the presence of VEGF (25 ng/ml). Although we have thoroughly tested native CRP previously and found no angiogenic response *in vitro*, we included it as a testing molecule for confirmation *in vivo*. Native CRP produced no notable angiogenic response (n = 3 and statistical comparison was not carried out with these implants) (Fig. 3B). In addition, using a Millipore cell permeability assay, we showed that incubation of a BAEC monolayer cultured on collagen with 10 μg/ml mCRP (8 h) induced a significant increase in cell permeability (p < 0.01), and similar to that produced by pre-incubation with 10% DMSO (Fig. 3C).

mCRP increased the expression of N-cadherin-often associated with inflammatory/unstable or aggressive angiogenesis (Fig. 3D); but no other cell surface markers of EC activation/cell junction remodelling (ICAM-1, VCAM, α_5_β_3_ integrin-data not included).

### *In vivo* mouse study

#### Treatment with mCRP significantly deteriorated non-cognitive and cognitive behavior of none-transgenic mice

Intrahippocampal injection of CRP distinctly deteriorated several aspects of general behavior and cognitive responses tested in NTg mice. As positive controls, five-month old 3xTg-AD mice showed changes to their behavior and their capacity of learning and memory, as expected^26^,^27^.

**Figure 4:**
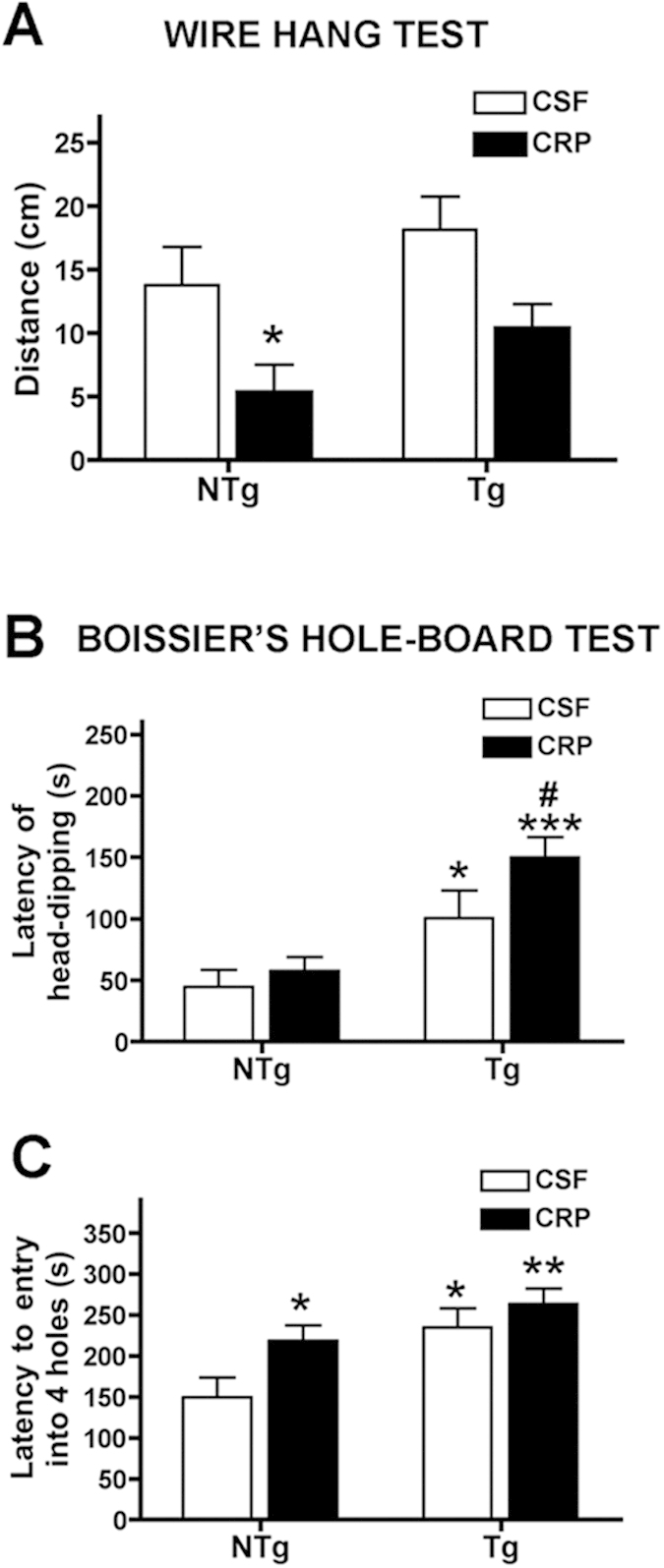
Behavioural changes observed following mouse-hippocampal injection of mCRP. Stereotactic injection of mCRP (50 μg) directly into the CA1 hippocampal region of mice were examined 3–4 weeks after operation. A significant reduction in balance and grip was see in non-transgenic animals in the presence of mCRP (wire hang test; (**A**) whilst head dipping latency in the Boissier’s hole-board test was significantly increased in 3xTg mice and further increased when mCRP was present (**B**). In the same test, the latency to entry into 4 holes was significantly increased in both mCRP-treated non-transgenic and 3xTg mice. Statistical analysis was done using two-way ANOVA (*P < 0.05).

mCRP did not decrease mouse reflex responses or coarse motor coordination on a rod, neither grip strength when clung on a wire (not shown). However, CRP reduced fine coordination significantly in NTg mice and as a trend in 3xTg mice, as shown in the wire hang test (Fig. 4A; p < 0.05). No significant changes were induced by CRP treatment on the freezing time, horizontal and vertical movements and emotionality behavior as tested in the open field test (not shown). CRP did not induce neophobia, anxiety or depression-like behavior in the NTg mice as tested in the corner test, dark and light box test and tail suspension test, respectively, nor increased the level of these behaviors in Tg mice (not shown). However, the treatment with CRP induced a significant effect in the animal behavior in the Boissier’s hole board test, significantly decreasing the exploratory activity of NTg mice and further decreasing that of Tg mice (Fig. 4B,C; p < 0.05).

**Figure 5:**
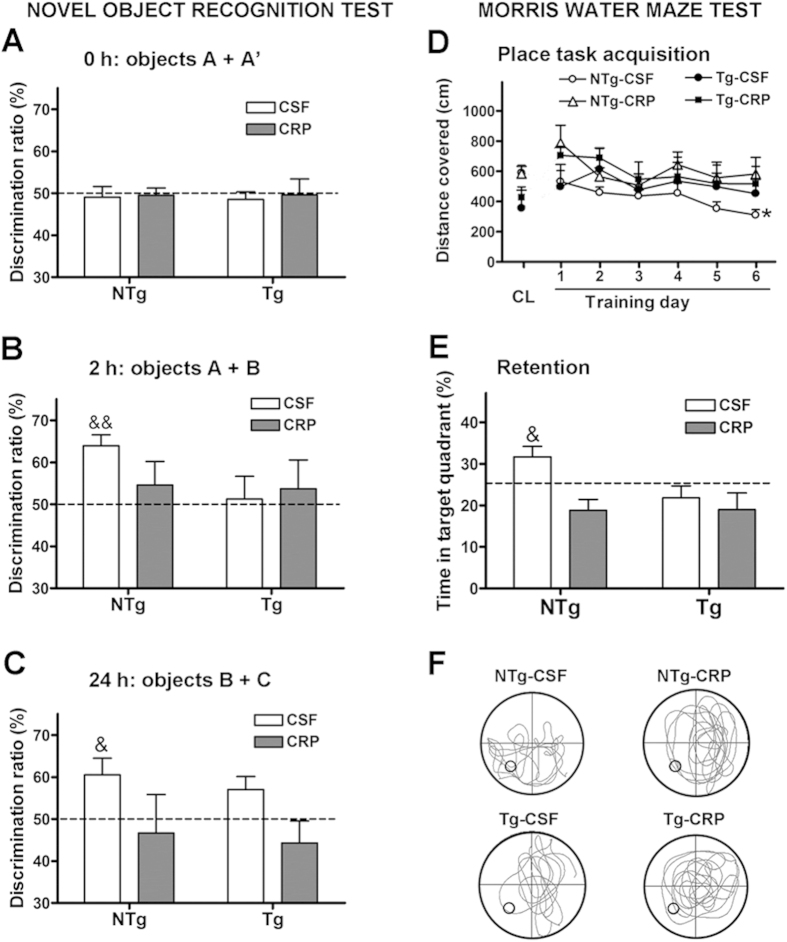
Cognitive effects of mCRP on mice following hippocampal injection. (**A**–**C**) shows results for novel object recognition. Whilst no effects were seen at time zero, a trend was seen after 2 h and after 24 h, visual discrimination ratio was significantly decreased in the presence of mCRP in both non-transgenic and 3xTg mice (**C**). Similarly, in the water maze test (**D**–**F**), mCRP-treated mice showed a significant increase in distance covered (**D**) and also reduction of % time in the target quadrant (**E**,**F**). Statistical analysis was done using two-way ANOVA (*P < 0.05).

mCRP induced cognitive loss in NTg mice, but cognition-related effects could not be detected in Tg mice because of their own low capacity of learning and memory. Lack of visual discrimination of a previously explored object induced by mCRP treated mice was detected in the novel object recognition test when object A was replaced by object B and C (Fig. 5A–C; p < 0.05). Significant reduced acquisition of spatial learning and lack of retention of memory induced by mCRP were demonstrated in the MWM (Fig. 5D–F; p < 0.05).

### Histology

**Figure 6:**
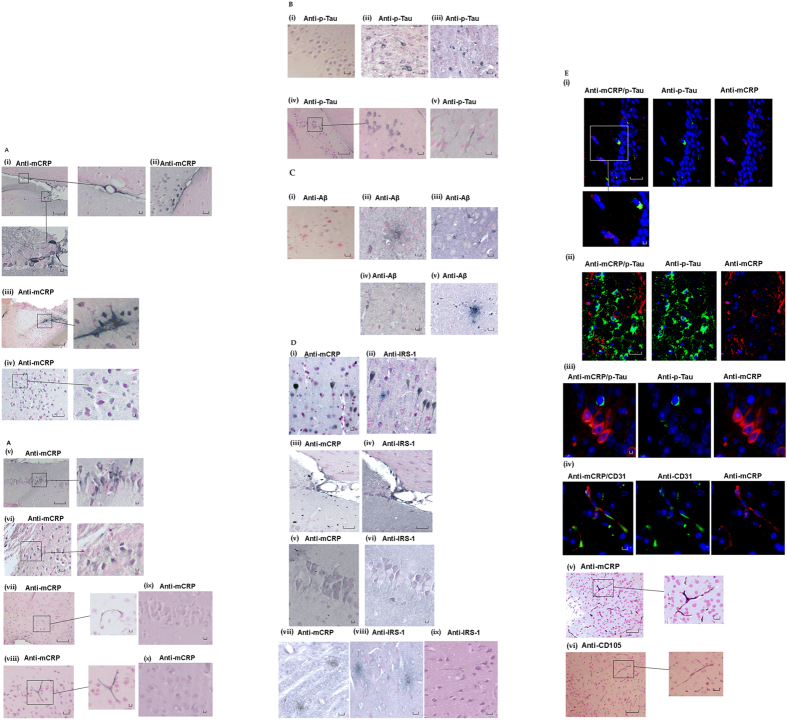
IHC of mouse brain tissue sections following mCRP hippocampal injection. Example staining results from histological and ICH analysis of mouse brain tissue sections (5 μ). (**A**) (i–iii), shows mCRP-positive ventricles (i), neurons close to the injection site (ii) and positive neuronal staining around cortical ventricular tracts (iii). (iv) There are numerous positively (peri-nuclear) stained irregular hypertrophic looking cortical neurons. In v, CA1 hippocampal neurons show strong mCRP-positivity whilst in vi, distant staining was observed in neurons of the hypothalamic region. In vii-viii, cortical microvessels are clearly stained for mCRP. ix shows non-transgenic control brain hippocampal neurons negative for mCRP staining and (x) shows cortical neurons also negatively stained for mCRP. (**B**) P-Tau positivity was increased in sections of cortical neurons. (i) shows an example of negative staining in hippocampal neurons of a control mouse whilst (ii) shows p-tau staining in cortical neurons from a 3xTg mouse and (iii) an equivalent section from an mCRP-injected animal. (iv) shows notable hippocampal (peri-nuclear) staining whilst (v) shows positive axonal cortical neuronal staining (v). Aβ staining is shown in (**C**) (i) shows negative staining in a normal non-injected mouse, ii and iii show plaque –like element staining and neuronal axons with peri-nuclear staining respectively, whilst iv and v show sections of 3xTg mice demonstrating a similar staining pattern. (**D**) shows p-IRS-1 and mCRP staining in cortical neurons and plaques (i-ii; serial sections) and matching areas of serial sections showing co-localization in ventricular tracts local to the injection site (iii-iv), CA1 hippocampal neurons (v-vi). vii-viii, shows the presence of p-IRS-1-labelled plaque-like mCRP-positive structures in the same region as mCRP positive areas. (ix) shows non-transgenic mouse cortical region negatively stained for p-IRS-1. (**E**) shows double immunofluorescent micrographs demonstrating Ca1 neuronal co-localization of mCRP (TRITC) and p-Tau (i-iii; plus insert), and in cortical microvessels (iv; CD31 = FITC). Areas of direct co-localization appeared yellow. Due to antibody binding restrictions, active angiogenic vessels were labelled with CD105 in serial sections and compared with mCRP staining. V-vi show mCRP and CD105 staining in the same vessels of serial sections respectively. No positively stained microvessels were observed in the normal non-transgenic mouse cortex (data not shown). Magnification bars 2.5 mm = × 400).

Following injection at CA1 hippocampal region, mCRP staining was identified along the retrosplenial ventral tract, lining of the dorsal 3^rd^ ventrical and within some surrounding major blood vessels and local cortical neurons (Fig. 6Ai) (n = 5) animals examined by histology in each group. All animals demonstrated the following similar features. Small microvessels were mCRP positive at the vicinity of injection site together with patchy areas of surrounding cortical neurons, especially in temporal association areas and primary auditory area, layer 5 and 6a. Scattered groups of neurons were mCRP-positive within cortex regions adjacent to injection site (Fig. 6Ai–iii arrows), and in more distant cortical areas (primary and secondary motor areas, layers 2–5; Fig. 6Aiv). There was a homogenous peri-nuclear staining in groups of neurons from the ipsilateral hippocampal region and cells appeared irregular and hypertrophic. The latter staining was more evident within hippocampal field CA1 pyramidal layer, dentate gyrus molecular and granular layers, but not in field CA3 (Figure Av) Interestingly, there was also notable mCRP staining within hypothalamic neurons, posterior hypothalamic nucleus, lateral hypothalamic area and periventricular hypothalamic nucleus (Fig. 6Avi).

Mouse cerebrum microvessels close to hippocampal formation were strongly positive for mCRP. Microvessels from local cortex regions were also positively stained (Fig. 6Avii-viii) Distally, staining within microvessels was evident within the hypophysis region. Non-injected control animals showed no positive staining for mCRP in hippocampal or cortical regions (ix and x respectively).

In order to assess AD pathology, we performed p-Tau and Aβ immunostaining on serial sections of mice brains. CA1 pyramidal neurons and dentate gyrus molecular and granular layers were positively stained for p-Tau and Aβ (Figs 6Bii,iii and 6Ciii respectively). There was significant p-Tau staining within the hippocampal CA3 region, shown positive for mCRP (Fig. 6Biv-vi), whilst Aβ was primarily increased in sporadic cortical neurons and diffuse plaque-like elements associated with dying neurons (Fig. 6Cii,iii), mimicking the pattern seen in 3xTg mice (Fig. 6Civ,v). P-Tau staining was generally more abundant within cortical areas, mainly in big pyramidal neurons both in ipsilateral and contralateral hemisphere, with notable axonal positivity. This was especially evident with ecthorinal area 5, layer 5 and temporal association areas, layer 6. Peri-nuclear and axonal staining was seen in piriform cortical neurons of coronal (Bregma −1.94 mm) sections in the hemisphere of injection. The later neurons were oedematous with ballooned, vacuolized morphology. Within ipsilateral basal ganglia, there were positive p-Tau neurons in thalamus, mainly posterior complex and posterior lateral nucleus of thalamus.

Since we identified an increase in p-IRS-1 within vascular endothelial cells on exposure to mCRP, we examined its expression and localization within our mCRP-treated animals by IHC. We identified increased p-IRS-1 expression within cortical neurons in the same areas of serial sections as mCRP-positive cells (Fig. 6Di,ii), as well as ventricular tracts (Fig. 6Diii,iv), hippocampal neurons (Fig. 6Dv,vi), some cortical microvessels and cortical plaques (Fig. 6Dvii,viii). No obvious staining was seen in cortical microvessels. Non-transgenic animals showed very little staining for p-IRS-1 (Fig. 6Dix).

Double IF labelling demonstrated co-immunolocalization of hippocampal neurons and CA1 and dentate gyrus neurons with mCRP (TRITC) and p-Tau (FITC) (Fig. 6Ei,ii respectively) as well as cortical microvessels mCRP (TRITC) and CD31 (FITC) (Figure 6Eiii).

Interestingly, serial sections from the same areas showed numerous mCRP positive medium sized microvessels that were also CD105-positive (Fig. 6Eiv,v). This was not seen in the contralateral hemisphere. Deep into the brain within periventricular areas of thalamus, including paraventricular nucleus of thalamus, lateral habenula, intermediodorsal nucleus and central medial nucleus of thalamus had medium sized microvessels that were CD105 positive. The density of the above positive blood vessels was higher closer to hippocampal dentate gyrus with postive neurons for mCRP and p-Tau on serial sections.

Analysis of negative control sections (normal wild-type mice injected with buffer only) showed very little staining of neurons for p-Tau or Aβ and no evidence of Aβ plaques as shown in the various images, whilst in contrast, positive control 3xT mice showed similar staining pattern to non-transgenic mCRP-injected animals but more widespread and more cortical diffuse staining with the appearance of amyloid plaques (See Fig. 6A–E control images).

### mCRP directly induced phosphorylation of potentially neurodegenerative signalling intermediates in cortical neurons

**Figure 7:**
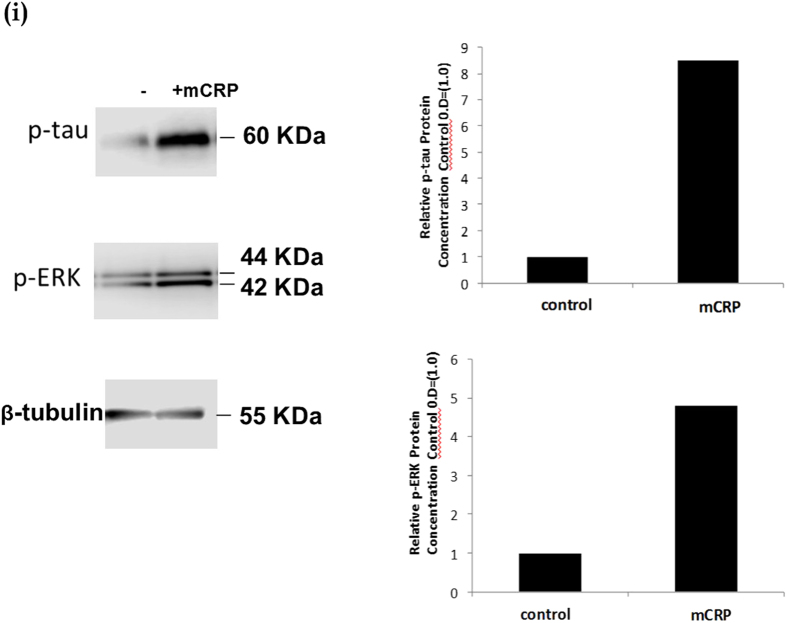
Western blotting showing mCRP-neuronal protein phosphorylation. Rat cortical neurons cultured in basal medium showed approximately an 8.5 fold increase in p-Tau expression and a 5-fold increase in p-ERK1/2 by Western blotting after 8 minutes treatment with mCRP (I-ii respectively; 10 μg/ml). In addition, (ii) mCRP induced increased phosphorylation of p-IRS-1 (5 fold), p-Akt (2.5 fold) and p-APP (2 fold). (iii) shows that pre-incubation with our anti-mCRP blocking antibody was able to inhibit mCRP signalling through p-ERK1/2 and p-Tau. These experiments were carried out at least twice and a representative example is shown. (iv) Kinexus phospho-protein Western array carried out on control neurons versus mCRP-treated cells (8 minutes; 10 μg/ml) revealed further proteins that could be involved in neuronal-mCRP signalling including focal adhesion kinase (2.2 fold increase) and p-53 (1.7 fold increase). Magnification bars 2.5 mm = ×400).

Western blotting demonstrated that rat cortical neurons cultured in basal medium for 24 h prior to experimentation, exhibited a notable increase in expression of p-Tau and p-ERK1/2 following 8 minutes of exposure to mCRP (10 μg/ml; Fig. 7i). In addition, p-IRS-1, p-AKT and p-APP were also increased in mCRP-treated cells (Fig. 7ii). Pre-treatment with blocking mCRP-antibody as described above before addition of mCRP notably reduced the phosphorylation of both p-ERK1/2 and p-Tau (Fig. 7iii). In order to gain a further understanding of possible signalling pathways activated by mCRP in neurons, we conducted a KINEXUS Western phospho-protein array and results from this indicated a possible increase in expression p-FAK, p38 and p53 whilst a reduction in expression of RSK-1 and ERK-5 was seen (Fig. 7iv). Further studies are required in order to identify the importance of these proteins in neuronal mCRP-induced signalling.

### The aggregation of Tau244–372 was induced directly by addition of mCRP *in vitro*

**Figure 8:**
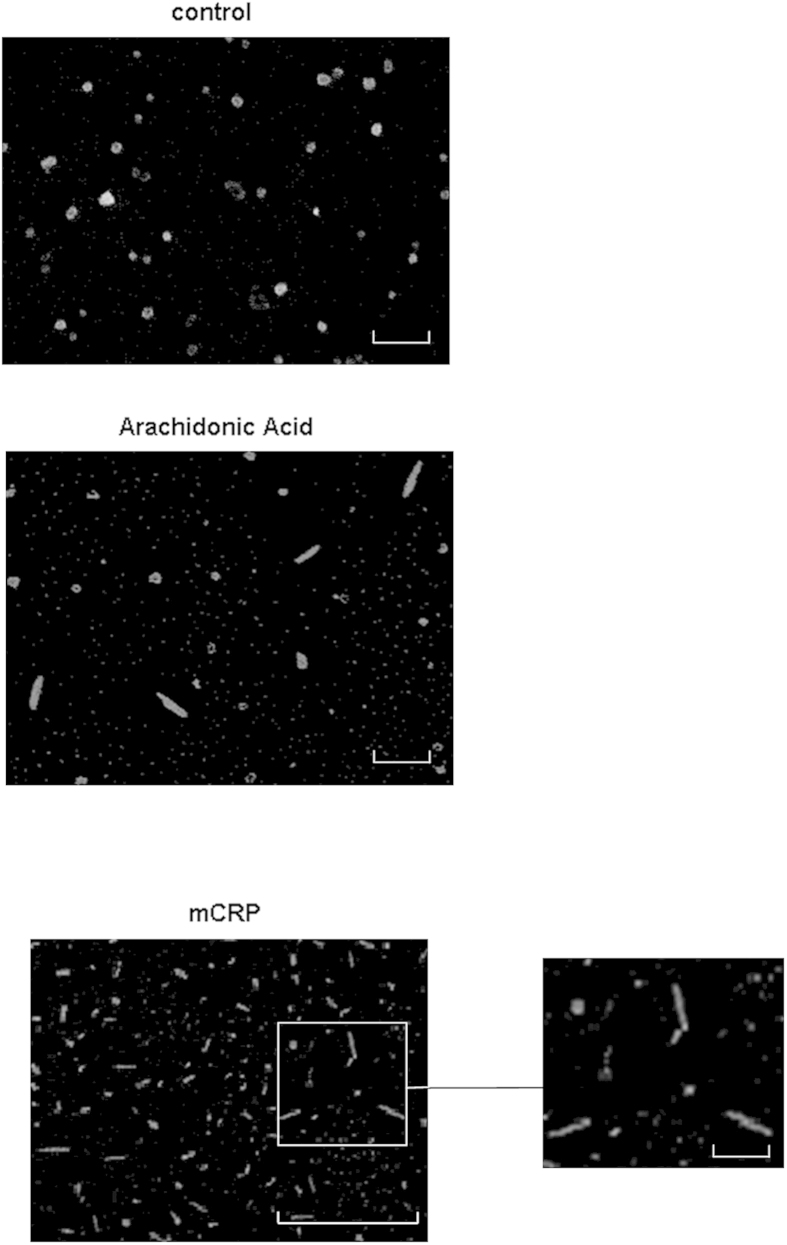
Tau fibrilization assay. *In vitro* assay showing Tau 244–372 aggregation induced by mCRP (10 μg/ml; 24 h) (**c**), with a similar profile to that produced by the positive control arachidonic acid (150 μM). (**a**) shows the control Tau incubated with all other buffer component’s minus CRP or arachidonic acid (**b**). These experiments were carried out at least twice and a representative example is shown. Magnification bars10 mm = 1 μM.

When Tau was incubated with mCRP (10 μg/ml) over a period of 24 h (time of incubation optimised from tests at 1 h-5 days in pilot studies not included) using a previously published protocol^28^, Scanning electron microscopical analysis of sputter-coated air-dried samples revealed increased helical tau filament polymerisation (dimerization) and formation to around 100 nm, whilst incubation with Arachidonic acid (150 μM), used as a positive control produced a similar effect (Fig. 8).

### Anatomopathological study

Localization and expression of mCRP in Alzheimer’s brain tissue with and without ischaemic stroke: Native CRP was almost un-detectable in any of our brain tissue samples (AD/AD with or without IS; data not included; see reference^29^ Slevin *et al*. 2010 for information on stroke patients).

There was strong expression of mCRP in the stroke brains from patients with clinically confirmed dementia and neuropathology of AD (β-amyloid-positive plaques and, tauopathy). mCRP was observed in peri-infarcted and infarcted regions in plaques from all five stroke patients with AD and IS (Supp. Fig. 1A; Table 1). mCRP positive neurons were also present in regions adjacent to the infarct but further away where the tissue appeared normal (i.e. no stroke tissue damage but positive for plaques and other features of neurodegeneration), the mCRP staining virtually disappeared. There was a weaker staining pattern within the infarcted core and particularly in old infarcts (14 days and beyond). Penumbral regions and other areas with strong tau/phospho-tau pathology showed the most intense staining for mCRP. The mCRP which as we demonstrated previously (8) becomes strongly expressed in ischaemic microvessels (Supp. Fig. 1B;), was also associated with neurons from within the plaques (Supp. Figure 1Bii) as well as with plaque elements with the appearance of neurofibrillary tangles (Supp. Fig. 1C). Interestingly, in AD patients (without known stroke), we observed frequent regions of tissue damage with the appearance of small cortical lacunes, and these regions stained most strongly for mCRP. mCRP-positive areas clearly surrounded neuronal plaques, but the staining was negative in end-stage dead neurons.

In sections from patients with clear AD pathology but no evidence of tissue damage caused from lacunar stroke or other hypoxic conditions, there was some staining of mCRP in neuronal plaques however there was no microvessel mCRP localization, suggesting further, that, mCRP deposition in neurons and microvessels are independent. The presence of mCRP in plaques maybe related to mCRP involvement with AD pathophysoiology and denovo synthesis and not only due to deposition from leaky microvessels in tissue infarcted areas.

Observation of microvessels from within the infarcted and peri-infarcted regions also showed an intense staining of mCRP (FITC green) in β-amyloid-positive (Rhodamine-red) capillaries (Supp. Fig. 1D; Table 1). Arrows show areas of co-localization in confocal images. The increased microvessel mCRP staining was present also in vessels without amyloid, but staining was more prominent in sections with severe amyloid angiopathy. Vessels expressing mCRP were almost always CD105-positive suggesting activation and perhaps the potential to undergo angiogenesis (IHC staining with anti-CD105-DAB brown and IF TRITC red using anti-mCRP antibodies; Supp. Fig. 1E). No expression of mCRP was seen in the contralateral hemispheres of patients following ischaemic stroke (data not included). Supp. Figure 2i shows similarity between mCRP staining of cortical neurons and in a serial section, tau phosphorylation of the same region in stroke-affected cerebral cortex. In Supp. Figure 2ii, lacunar stroke micro-infarct core has been identified (arrow) and a gradual reduction in intensity of mCRP staining is seen as we move away from the damaged tissue region. This was common to all similar regions we examined.

Supp. Figure 3 shows a novel signalling pathway through which mCRP may contribute to pathological development of dementia. Key novel elements include IRS-1, and NCAM.

## Discussion

Epidemiological studies have demonstrated that approximately 25% of elderly patients show signs of dementia within 3 months of ischaemic stroke. Desmond *et al*.^1^ demonstrated a significant increased risk of dementia associated with ischaemic stroke with specific relationships to cerebral hypoxia/ischaemia. As mentioned earlier, ischaemic stroke exacerbates dementia in Alzheimer´s patients and animal models have demonstrated a strong relationship between neuroinflammation, increased platelet activation (which could involve mCRP^30^—; and AD/stroke toxicity^4^,^31^. In these cases, hypoxia is often associated with small vessel disease and vessel constriction or in-patency (vascular dementia; CAA). Vascular remodelling is a key feature of the neurodegenerative process, abnormal angiogenesis being strongly associated with β-amyloid deposition and the presence of NFTs in a study of post-mortem brain samples from AD patients, suggesting a relationship with tissue injury^32^. Similarly, Meyer EP *et al*.^33^ showed using APP23tg mice and vascular casting, that vasculature often ended (was blocked) at the sites of developed amyloid plaques and surrounding hypoxic regions had tried to compensate by eliciting angiogenesis.

Since circulating native CRP levels increase dramatically during cardio/cerebrovascular events and mCRP is now known to become tissue and cell associated as a monomer with strong biological properties^7^,^10^,^30^,^34^, it made sense to investigate if it could be involved in modulating cell signalling associated with plaque development or vascular damage associated with small vessel disease in AD affected individuals. In this regard, we can only measure circulating pCRP concentrations (as pCRP is not found associated with tissue/cells), which as stated previously range during active infection and prior to/during cardio/cerebro-vascular ischaemia between 40–200 μg/ml in patients^7^. And hence our chosen mCRP delivery of between 5–10 μg/ml (*in vitro*) and-50 μg/total drip- (1 μg/minute) delivery comfortably fits within these limits and was designed to reflect this following our previous work and that of others showing no cytotoxicity to cells within this range of use^8^,^9^,^10^.

In addition, our previous histological studies of ischaemic stroke patients allowed us to identify the expression of mCRP in co-existing amyloid plaques and elements with the appearance of NFTs; however, where there was no evidence of previous ischaemic stroke or in regions of lacunar/silent strokes, very little mCRP was seen. In patients with AD but without evidence of stroke, mCRP was only evident by its relatively weak incorporation into some AD plaques neighbouring damaged neurons, suggesting an additional possible de novo mechanism of synthesis distinct from deposition through vascular leakage. Native CRP was not found to be expressed in any of the tissue samples^8^.

Here, we demonstrate a potentially key role for mCRP in patients with previous vascular or ischaemic brain damage (which could include brain trauma injury) in perpetuating dementia onset. Our four key elements for proof of principle are 1) mCRP induces abnormal angiogenesis, producing vessels *in vitro* allowing, greater permeability, and signalling activation reflecting a possible mechanism for perpetuating inflammation; 2) CA1 hippocampal injection of mCRP in a murine model of AD, directly induced cognitive and behavioural decline concomitant with AD-like brain structural changes including increased expression of p-Tau and β-amyloid plaque production; 3) mCRP induced tau filament polymerization *in vitro* and in addition, mCRP induced neuronal signalling pathways associated with AD pathological development; 4) a detailed histological study in patients with AD with and without stroke demonstrated a strong expression and co-localization of mCRP and associated signalling molecules with AD elements, with strong co-localization to areas of tissue disruption caused by infarct.

In evidence for the impact of mCRP on vascular function, we showed using tri-dimensional spheroids in combination with TEM that inter-cellular spaces or gap junctions between adjoining cells were notably greater in sprouts developing from mCRP-treated tri-dimensional spheroids. In addition, mCRP significantly increased the permeability of a confluent, barrier endothelial cell monolayer to FITC dextran and also produced haemorrhagic tissue lesions concomitant with angiogenesis following dorsal matrigel implantation *in vivo* (examined macroscopically only). This data suggests strongly that mCRP may induce an increased permeability of abnormally developing microvessels after tissue injury, and this could be linked to exacerbated inflammation and/or haemorrhage in the region if the same pattern were reproduced *in vivo* in developing or damaged microvessels. This could have relevance to vascular dementia and that linked to ischaemic stroke, where, the micro-environment existing in the vicinity of susceptible vessels may be unbalanced in the presence of mCRP leading to more aggressive but less efficient angiogenesis producing the same immature and weak vessel walls seen in tumour/plaque vascularisation^33^,^35^,^36^.

We then investigated the potential signalling mechanisms through which mCRP might affect vascular formation and development using a specifically designed Western phospho-protein screening array with a particular focus on vascular activation pathways associated with neurodegenerative disease. Quantitative analysis of protein phosphorylation changes induced in BAEC in the presence of mCRP demonstrated that p-IRS-1 and p-tau amongst others were notably increased. Insulin growth factor and its substrates IRS-1/2 have been implicated in the vascular complications of diabetes associated with AD in association with β-amyloid clearance; however the exact role and mechanism are not yet understood^37^. Here, we show that siRNA knock-down of IRS-1 partially inhibited the pro-angiogenic effects of mCRP by blocking tube-like structure formation in matrigel and abnormal, fragile sprout formation in EC-generated spheroids. IRS-1 is now known to be strongly pro-angiogenic, and down-regulation of its expression blocks angiogenesis both *in vitro* and *in vivo*^38^. Hence, this is one potential novel mechanism that mCRP may try to promote new vascular growth in angiogenic areas of damaged or stroke-affected areas of AD brain tissue^39^. It is interesting to consider that the insulin-like growth factor-1 receptor (IGF-1R) might be a candidate receptor for mCRP binding since it directly phosphorylates IRS-1 and its inhibition has also been shown to be sufficient to block angiogenesis.

We also noted that mCRP had the ability to phosphorylate tau on serine 214. Recently, Zhu *et al*.^29^ demonstrated that in vascular ECs, phosphorylation of non-neuronal tau at serine 214 was associated with abnormal microtubule organization. They further described how this activated form of tau induced intracellular gaps and increased macromolecular permeability in a PKA/cAMP dependent mechanism ultimately disrupting the EC barrier. In addition, Balczon, *et al*.^40^ showed that pseudomonas aeroginosa exotoxin Y was able to hyper-phosphorylate tau in pulmonary microvascular ECs and that this disrupted microtubule assembly, potentially leading to increases vascular permeability, and barrier breakdown/oedema *in vivo*. In addition, we showed that mCRP induced an increase in expression of NCAM-a marker of immature endothelial cells and linked to active increased EC permeability^41^, but did not affect VCAM. ICAM or integrin expression. Therefore, Tau phosphorylation/NCAM activation could be a mechanism through which mCRP increased the EC permeability. Note, our previous work and others clearly demonstrated an almost complete lack of biological signalling activity relating to angiogenesis and inflammation of pCRP^8^,^30^.

Since mCRP was able to phosphorylate tau, we examined other pertinent candidates linked to neurodegenerative progression and identified increased expression of both amyloid 1–42 (24 h) and p-PEN-2/p-APP within 8 minutes of treatment. This is not the first report of β-amyloid-cleavage in EC. This phenomenon has been reported recently by Rajadas *et al*.^42^ in Hep-1 cells associated with enhanced APP expression and linked to endothelial and vascular abnormality^34^. Therefore, de novo modification/production of toxic amyloid could also be related to mCRP-induced endothelial dysfunction *in vitro*, *in vivo*, and potentially in vascular-based dementia. We investigated if the antibody to mCRP could potentially block the down-stream signalling activation and found that pre-incubation with anti-mCRP did significantly reduce angiogenesis and also signalling through p-ERK1/2 and p-IRS-1 with an ND_50_ value of around 1 μg/ml (not kinetically determined).

Further evidence of a possible role of mCRP in AD has been provided by Strang *et al*.^11^ who recently showed that Aβ plaques generated *in vitro* were able to dissociate native CRP to mCRP. In addition, cortical plaques from patients with AD were positive for mCRP whilst nCRP was not present, suggesting a strong link to AD pathology and that mCRP could be a driving feature of plaque development.

Bi *et al*.^43^ suggested that intra-vascular pCRP injection in mice resulted in increased expression of APP and presenilins. However, they were not able to determine the fate of CRP, its possible conversion to the monomeric form, nor activation of these proteins. In addition the use of the murine model which does not respond to inflammatory stimulus by increasing CRP is an unusual model to apply to this protein, and hence this work, although mentioned in passing, may not provide any significant advances to our knowledge.

Investigating this possibility further, we showed that direct hippocampal injection of mCRP in mice resulted in both CA1-3 positively stained neurons as well as local microvascular staining. Neuronal cells stained concomitantly with p-Tau and cortical regions of microvessels positive for mCRP were also CD105 and p-IRS-1-positive (serial section analysis) suggesting a promotion of angiogenesis or vascular activation. mCRP became ‘stuck’ indefinitely in the ECM and was found in cortical, hippocampal and hypothalamic neurons, producing vacuolated and/or swollen cells (present and remaining more than 1-month after injection). No overall cell loss was observed, and control mice injected with equivalent concentrations of CSF protein showed no reaction nor signalling activation. Tau aggregates were seen localised to abnormal looking neurons and axons also became p-Tau-positive. In addition, β-amyloid-like plaques were present and positively stained for mCRP, and overall the pattern of pathological staining was not dissimilar to that produced in transgenic animals visualised by IHC/histology. Most importantly, animals showed behavioural and cognitive deficits similar to triple transgenic animals with induced progressive AD-like brain pathology, including novel object recognition failure, Morris water maze distance and time, and wire-hang testing^44^. This provides the first indirect evidence that mCRP laid down within the brain parenchyma (such as following brain infarction) might directly promote development of neurodegeneration. No further cognitive or behavioural decline was observed in 3xTg animals that were also injected with mCRP. Since the level of decline in these animals is quite severe (and significantly more than that seen in mCRP-injected only animals) this was perhaps to be expected. In addition, mCRP was not detected in normal nor 3xTg mice even as a consequence of or concomitant with neurodegenerative pathway activation, suggesting it is not produced and/or deposited in this model as part of a normal inflammatory cascade, nor produced by de novo synthesis within the brain.

Since mCRP was found within neurons, we examined the effects of this molecule on cortical neuronal signalling using Western blotting and Kinexus phospho-microarrays. Similar to ECs, mCRP induced an increase in p-ERK1/2 and p-Tau expression, and in addition, p-APP, p-Akt and p-IRS-1 were stimulated within 8 minutes exposure. Once again, blocking mCRP-antibody incubation was sufficient to inhibit p-ERK1/2 and p-Tau signalling. Additional proteins increased on the microarray included focal adhesion kinase (FAK) and p53, both of which could influence signalling linked to Aβ-induced cellular apoptosis, although further characterization of their importance would need to be carried out^45^. Similarly, Aβ-induced JNK-IRS-1 signalling is also implicated in insulin resistance in diabetes and to pathological progression of AD^46^.

One of the hallmarks of AD is the fibrillization of tau protein wherein, individual monomers aggregate, particularly when hyper-phosphorylated eventually to form paired helical filaments^47^ and this can be visualised using S.E.M./T.E.M^48^. In our study, we showed that tau oligomers produced evidence of tau filament assembly/aggregation in the presence of mCRP, and similar to that induced by arachidonic acid used as a positive control. We also identified similar looking mCRP-positive staining fibrils within our murine-mCRP-injected brainsa and AD patients with stroke. Several studies have identified a correlation between neurotoxicity and cognitive dysfunction, tau aggregate expression preceding NFT formation in animal models^49^, which leads to a potential hypothesis that mCRP could contribute to this pathological progression.

Interestingly, Thiele *et al*.^50^ recently showed that native CRP dissociating to mCRP in a rat intravital model of lipopolysaccharide-induced cremasteric muscle inflammation, significantly enhanced leukocyte rolling, adhesion and transmigration. In addition, stabilising the native CRP by blocking its dissociation using 1,6-bis(phosphocholine)-hexane, abrogated these effects. In our work, we specifically chose the murine model as under inflammatory conditions, CRP production is a minor part of the acute response to insult-and was not evident within the brains of our transgenic AD mice. Although we did not perform stereotactic injection of native CRP in our model, it is likely that it would have undergone a similar fate within the brain on contact with cells and tissue dissociating to mCRP and producing a similar effect to the mCRP-this would be our hypothesis of how the majority of mCRP may build up within the damaged brain tissue in AD. Further studies should be carried out to detail this. A limitation of the use of the murine model here, is that it cannot be used effectively to identify the impact of mCRP for example after stroke or vascular injury/traumatic brain injury on pathophysiological AD progression, due to its lack of production as an acute phase response protein. A CRP over-expressing animal combined with MCAO or other injury model could be used to give a better indication of its role (i.e. +/− mCRP in the brain)^51^ although larger mammalian models would have to be used in order to accurately depict a role in chronic neuro-deterioration e.g. the canine model that would provide proof-of-concept leading potentially to clinical trials^52^.

In AD patients, we showed that expression of mCRP, but not native CRP was expressed strongly in microvessels but only following ischaemic stroke and in stroke-affected regions. Vessels were also β-amyloid positive in many cases suggesting the presence of small vessel disease and usually CD105-positive suggesting abnormal activation and perhaps angiogenesis^33^,^41^. Cerebrovascular pathology is thought to be a key element associated with AD pathology and in particular, inhibition of angiogenesis, which may be an attempt to re-perfuse hypoxic areas of brain tissue, which may be attributed to β-amyloid deposition^4^. Strong mCRP positivity was observed within plaques-again particularly in patients with ischaemic stroke, and in addition, neurons and NFTs in p-tau/Aβ strongly positive regions were also stained positive for mCRP. It is interesting that when we examined micro-infarcted areas or lacunar strokes from patients with AD, there appeared to be a gradient of expression of mCRP being strongest near to the infarct core decreasing to a weak expression approaching regions with the appearance of normal tissue structure. This was concomitant with a greater concentration of p-Tau staining and Aβ-positive material.

Therefore, our hypothesis here is that mCRP may provide a causative link between ischaemic stroke, micro-infarction/lacunar insult or traumatic brain injury associated with vascular damage and inflammation,- and the significantly increased risk of development of dementia experienced within this population-based on the findings described above. As there are currently no effective and specific mCRP-inhibitors, production of such as small molecule inhibitors/antagonists or blocking antibodies could form the basis of a novel therapeutic strategy to inhibit down-stream processing linked to neurodegeneration/dementia after stroke.

## Additional Information

**How to cite this article**: Slevin, M. *et al*. Monomeric C-reactive protein-a key molecule driving development of Alzheimer’s disease associated with brain ischaemia? *Sci. Rep*. **5**, 13281; doi: 10.1038/srep13281 (2015).

## Supplementary Material

Supplementary Information

## Figures and Tables

**Table 1:** Patient details of Ischaemic stroke samples +/− AD from Belvitge Hospital.

Patient Number	IS/AD	AGE	SEX	Time of death alter IS	GDS-Fast/CDR	Hachinski scale	Plaque expression of nCRP	Plaque Expression of mCRP	Microvessel Expression of nCRP	Microvessel Expression of mCRP
1	AD	84	M	—	6A	1	—	—	+—	—
2	AD	84	M	—	7B	1	—	+	—	+
3	AD	77	M	—	5	2	—	—	—	—
4	AD	84	M	—	7C	1	—	+	+—	+—
5	AD	87	F	—	6B	0	—	+—	—	+
6	AD/IS	63	F	2	4	9	—	+++	—	+++
7	AD/IS	51	M	3	3	8	—	+++	+	+++
8	AD/IS	51	M	9	3	10	—	+	+	+++
9	AD/IS	68	M	19	5	8	—	++	+—	++
10	AD/IS	75	M	29	6D	11	—	+	—	++

**Table 2:** AD patient/specimen details from Bristol Brain Bank, UK.

BB NO	MRC ID	DIAG 1	DIAG 2	DIAG 3	AGE	SEX	Braak Stage	PM DELAY	APOE
119	BBN_8748	AD	NO	NO	91	F	6	78	3.4
217	BBN_8846	AD	NO	NO	84	F	6	96	4.4
221	BBN_8850	AD	NO	NO	84	F	5	41	4.4
270	BBN_8899	AD	NO	NO	92	M	5	45	2.4
317	BBN_8945	AD	NO	NO	91	F	4	12	4.4
341	BBN_8969	AD	NO	NO	95	M	3	48	3.3
434	BBN_9060	AD	NO	NO	87	M	n/a	12	n/a
451	BBN_9076	AD	NO	NO	84	F	5	20	3.4
456	BBN_9081	AD	Argyrophilic grain disease	NO	92	M	2	84	n/a
465	BBN_9090	AD	CAA	NO	83	M	6	20	3.3
496	BBN_9120	AD	NO	NO	84	F	n/a	24	2.3
530	BBN_9154	AD	NO	NO	81	M	n/a	28	3.4
677	BBN_9255	AD	NO	NO	81	M	n/a	16	n/a
691	BBN_9269	AD	NO	NO	83	M	4	99	3.4
697	BBN_9275	AD	NO	NO	87	M	6	36	4.4
731	BBN_9309	AD	NO	NO	88	F	4-5	88	3.3
745	BBN_9323	AD	NO	NO	84	F	6	20.5	2.3
760	BBN_9338	AD	CAA	SVD	95	M	4	27	3.4
768	BBN_9346	AD	NO	NO	88	F	6	64	3.3
821	BBN_4232	AD	CVD	NO	84	F	6	65.5	n/a
160	BBN_8789	CONTROL	NO	NO	78	F	2	24	n/a
323	BBN_8951	CONTROL	NO	NO	96	F	n/a	n/a	n/a
721	BBN_9299	CONTROL	NO	NO	90	M	2	5.5	2.3
733	BBN_9311	CONTROL	NO	NO	93	M	3	37.75	2.3
751	BBN_9329	CONTROL	NO	NO	80	M	0	45.75	3.3
762	BBN_9340	CONTROL	NO	NO	94	F	2	21	2.3
781	BBN_4205	CONTROL	NO	NO	87	M	2	24	3.3
786	BBN_9354	CONTROL	NO	NO	85	M	2	30.5	3.3
818	BBN_4229	CONTROL	NO	NO	87	F	3	47	2.3
826	BBN_9365	CONTROL	NO	NO	86	F	2	32	3.4

The AD cases all had a history of progressive dementia and were selected on the basis of a diagnosis according to CERAD of ‘definite AD’^53^ and a Braak tangle stage of V-VI^54^; according to NIA-Alzheimer’s Association guidelines^55^, AD neuropathological change was considered a sufficient explanation for the dementia in all cases. The normal controls had no history of dementia, few or no neuritic plaques, and no other neuropathological abnormalities.
