# Supplementary material for: Monomeric C-reactive protein-a key molecule driving development of Alzheimer’s disease associated with brain ischaemia?
Source: Sci Rep. 2015 Sep 3;5:13281. doi: 10.1038/srep13281 (PMC4558604; doi:10.1038/srep13281)
Supplement: Supplementary Information [file srep13281-s1.pdf]

Monomeric C-reactive protein-a key molecule driving development of Alzheimer's disease associated with brain ischaemia?

Slevin M, Matou S Zeinolabediny Y Corpas R Weston R, Liu D Boras E, Di Napoli M Petcu E, Sarroca S Popa-Wagner A, Love S Font M.A Potempa L.A Al-baradie R Sanfeliu C Revilla S Badimon L Krupinski J

Supp. Figure 1: Immunohistochemistry showing localization of mCRP i-ii) in the regions containing β-amyloid-positive plaques (iii) of patients with AD following stroke (Ai-Aiii; arrows; patient 6). Expression can also be seen at the higher magnification in affected neurons (x 200). Figure 1B shows strong mCRP staining in microvessels and early stage neuritic plaques from patient 6 who had suffered previous ischaemic stroke (Bi-Bii; arrows; x 40) and Biii shows a cortical region near to the infarcted zone that is strongly positive for mCRP in microvessels and plaques. mCRP appeared to stain NFTs in these regions (Ci-ii) whilst a strong co-localization of mCRP (TRITC) with Aβ (FITC) (D) and CD105 (DAB)-suggesting angiogenesis (E). Magnification bars 2.5mm= x 400).

Supp. Figure 2: Patient 8, (i) serial sections of stroke-affected cortex showing an almost identical pattern of staining with mCRP antibody (top) and p-Tau (bottom). Magnification x 200. (ii) Patient 6 low magnification of mCRP staining (DAB) showing the micro-infarct localization (arrow) and curved lines demonstrating as we move further away from the core, the mCRP staining becomes weaker and weaker. Magnification bars 2.5mm= x 400).

Supp. Figure 3: Shows a schematic highlighting probable novel and key signalling intermediates associated with mCRP-cell interactions which could contribute to development of vascular dementia/AD.

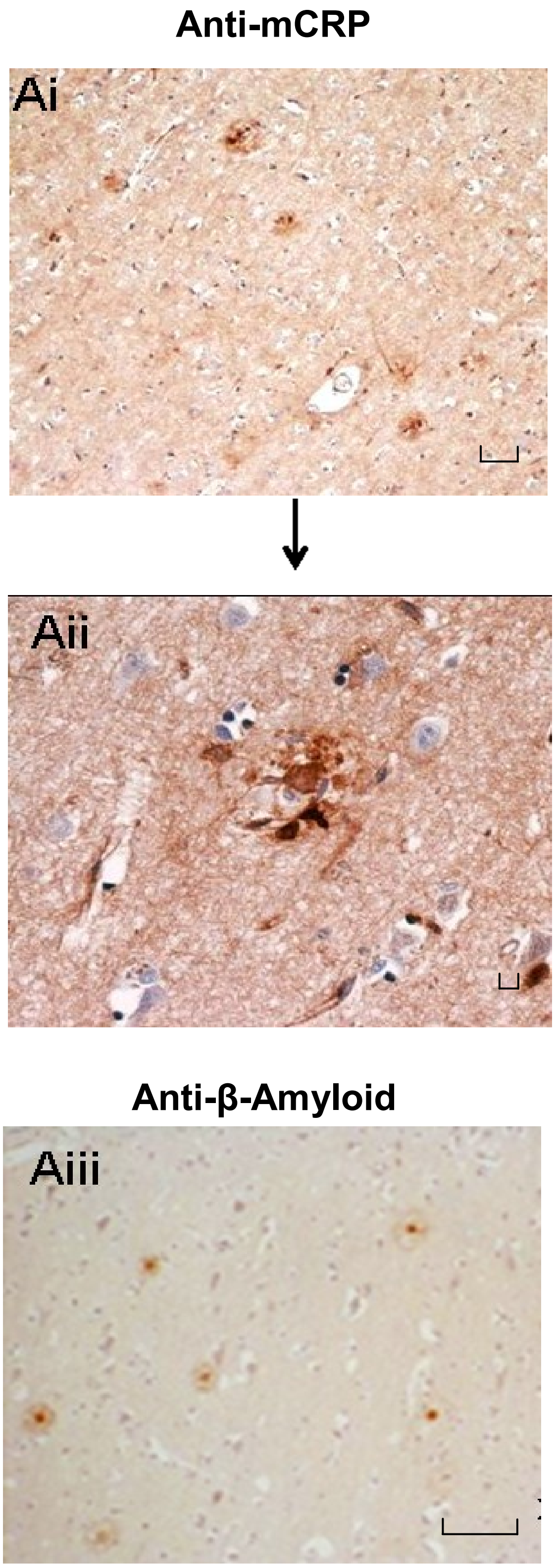
Anti-mCRP
Ai
Aii
Anti-β-Amyloid
Aiii

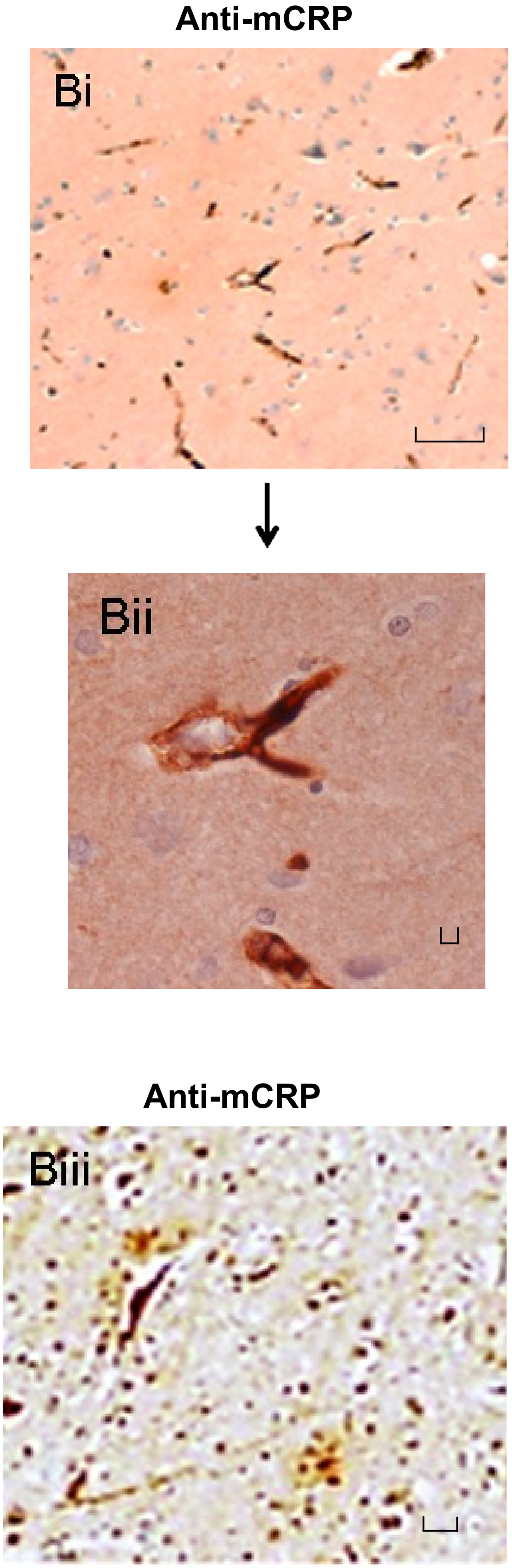
Anti-mCRP
Bi
Bii
Anti-mCRP
Biii

Anti-mCRP

Anti-mCRP

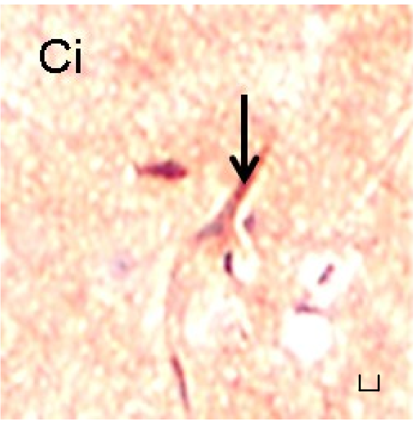

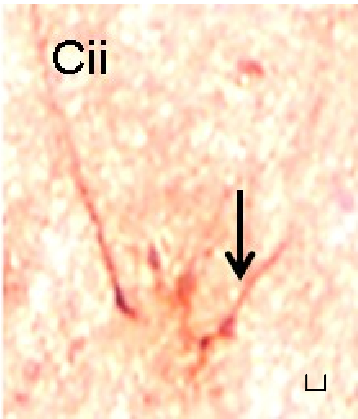

Anti-mCRP/Aβ

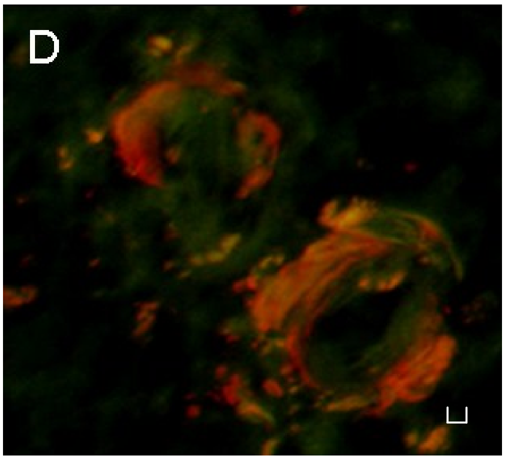

Anti-mCRP/CD105

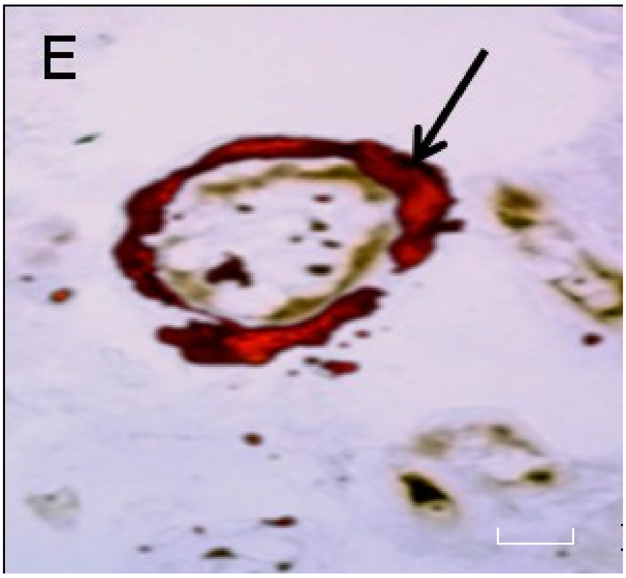

(i)

Anti-mCRP

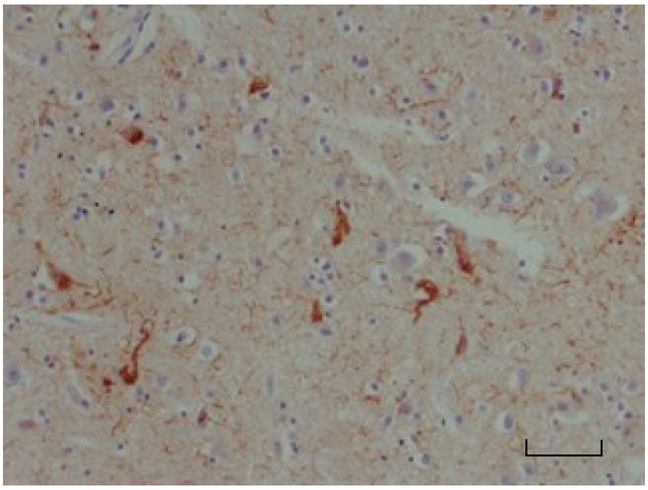

Anti-p-Tau

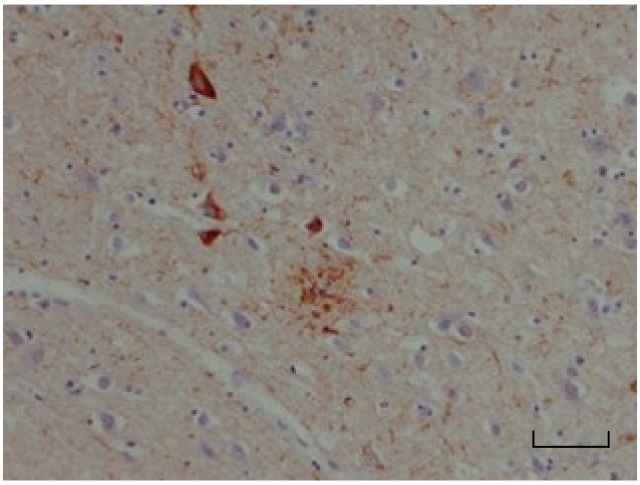

(ii)

Anti-mCRP

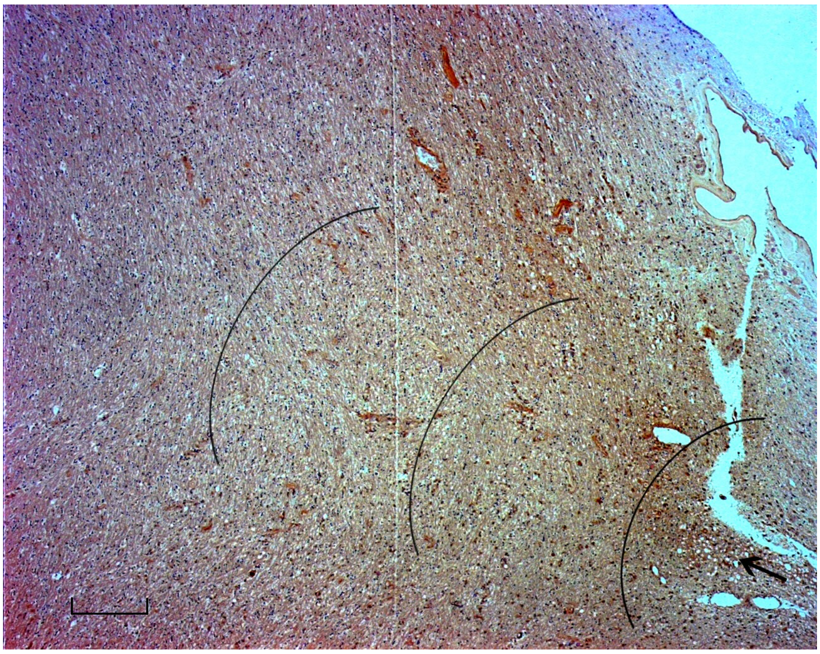

Anti-mCRP

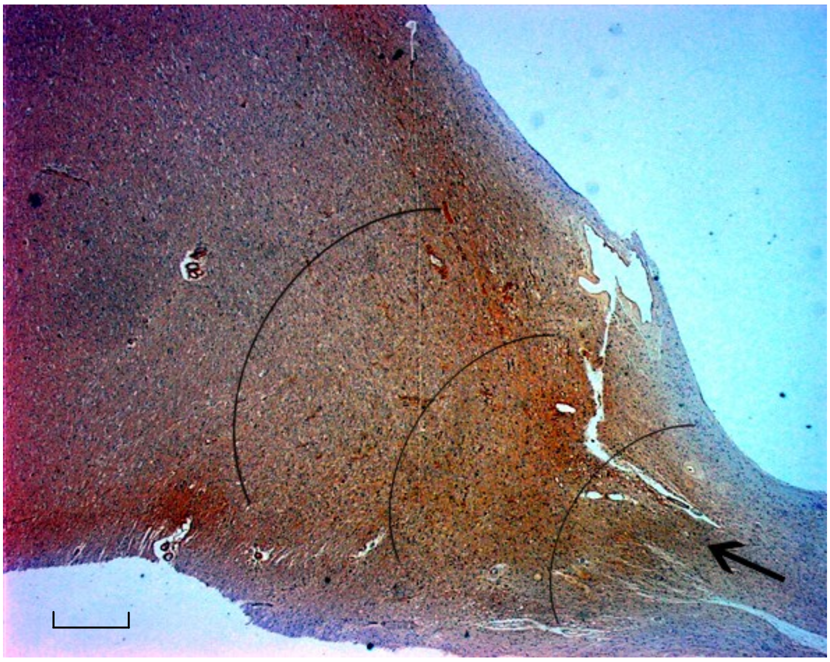

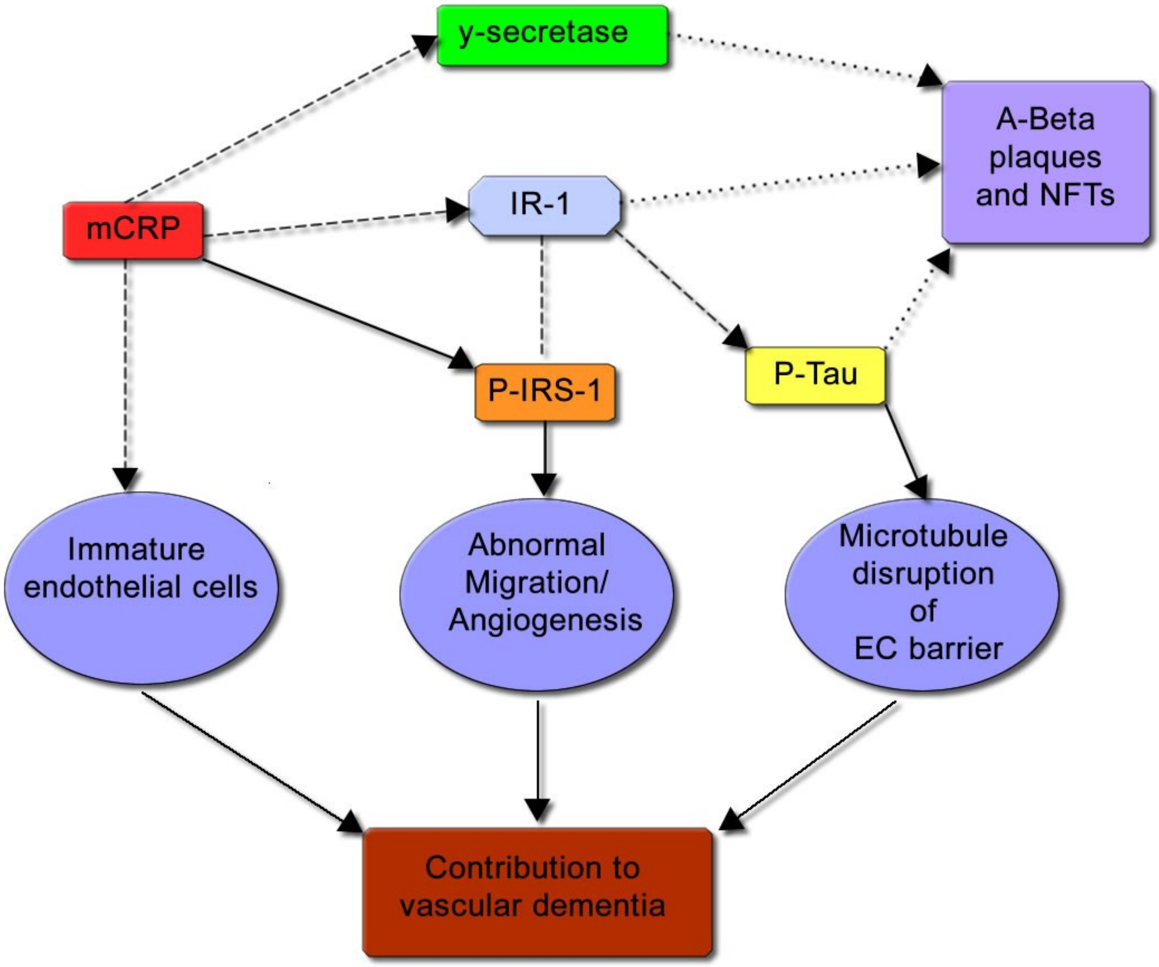
y-secretase
A-Beta plaques and NFTs
IR-1
mCRP
P-IRS-1
P-Tau
Immature endothelial cells
Abnormal Migration/ Angiogenesis
Microtubule disruption of EC barrier
Contribution to vascular dementia
